# Auxin‐activated MdARF5 induces the expression of ethylene biosynthetic genes to initiate apple fruit ripening

**DOI:** 10.1111/nph.16500

**Published:** 2020-03-28

**Authors:** Pengtao Yue, Qian Lu, Zhi Liu, Tianxing Lv, Xinyue Li, Haidong Bu, Weiting Liu, Yaxiu Xu, Hui Yuan, Aide Wang

**Affiliations:** ^1^ College of Horticulture Shenyang Agricultural University Shenyang 110866 China; ^2^ Liaoning Institute of Pomology Xiongyue 115009 China

**Keywords:** apple, ARF5, auxin, DNA methylation, ethylene, fruit ripening

## Abstract

- The gaseous plant hormone ethylene induces the ripening of climacteric fruit, including apple (*Malus domestica*). Another phytohormone, auxin, is known to promote ethylene production in many horticultural crops, but the regulatory mechanism remains unclear.
- Here, we found that auxin application induces ethylene production in apple fruit before the stage of commercial harvest, when they are not otherwise capable of ripening naturally.
- The expression of *MdARF5*, a member of the auxin response factor transcription factor (TF) family involved in the auxin signaling pathway, was enhanced by treatment with the synthetic auxin naphthaleneacetic acid (NAA). Further studies revealed that MdARF5 binds to the promoter of *MdERF2*, encoding a TF in the ethylene signaling pathway, as well as the promoters of two 1‐aminocyclopropane‐1‐carboxylic acid synthase (ACS) genes (*MdACS3a* and *MdACS1*) and an ACC oxidase (ACO) gene, *MdACO1*, all of which encode key steps in ethylene biosynthesis, thereby inducing their expression. We also observed that auxin‐induced ethylene production was dependent on the methylation of the *MdACS3a* promoter.
- Our findings reveal that auxin induces ethylene biosynthesis in apple fruit through activation of *MdARF5* expression.

## Introduction

Ripening is a key physiological process during fruit development, and is usually characterized by an increase in sugar content, and changes in fruit color, firmness and texture (Klee & Giovannoni, 2011). Based on differences in the production of the hormone ethylene during ripening, fruit are classified as ‘non‐climacteric’, with low ethylene production, and ‘climacteric’, with a rapid increase in ethylene at the onset of ripening (Adams‐Phillips *et al.*, 2004). Two ethylene systems are thought to exist in plants: System I ethylene is produced in vegetative tissues including young fruit, while System II ethylene is produced predominantly during ripening of climacteric fruit (McMurchie *et al.*, 1972; Alexander & Grierson, 2002). Production of ethylene is required for climacteric fruit ripening, and reducing its production can significantly delay the ripening process (Lohani *et al.*, 2004).

Ethylene biosynthesis consists of two steps: the ethylene precursor SAM (*S*‐adenosyl methionine) is first converted to ACC (1‐aminocyclopropane‐1‐carboxylic acid) by ACC synthase (ACS) and ACC is then oxidized to ethylene by ACC oxidase (ACO) (Adams & Yang, 1979). Ethylene is perceived by its receptors and the ethylene signaling pathway is then activated via the EIN3/EIL (ethylene insensitive 3/EIN3 like) transcription factor (TF), which in turn activates ERF (ethylene response factor) secondary TFs that regulate the expression of ethylene responsive genes (Guo & Ecker, 2004).

Regulation of ethylene biosynthesis has been widely studied in fruit. For example, silencing of tomato (*Solanum lycopersicum*) *SlACS2*, which is crucial for System II ethylene biosynthesis, significantly suppresses ethylene production and fruit ripening (Oeller *et al.*, 1991; Barry *et al.*, 2000), and suppressing the expression of tomato *SlACO1* has a similar outcome (Picton *et al.*, 1993). In apple, three *ACS* genes are expressed at different stages of fruit development and ripening: *MdACS6* expression is initiated immediately after the day of full bloom; *MdACS3a* expression is initiated around 105 d after full bloom in cv Golden Delicious; and *MdACS1* is specifically expressed during ripening (Wang *et al.*, 2009; Li *et al.*, 2015). Moreover, *MdACS1‐* and *MdACO1‐*silenced apples were found to produce very low levels of ethylene production during ripening (Dandekar *et al.*, 2004; Schaffer *et al.*, 2007). Ethylene biosynthesis has been shown to be transcriptionally regulated in fruit. For instance, in tomato, the MADS‐box family TF RIPENING INHIBITOR (RIN) regulates *SlACS2* by binding to its promoter (Ito *et al.*, 2008), while in banana (*Musa acuminata*) the MaNAC1/2 protein interacts with MaEIL5, resulting in decreased ethylene production (Shan *et al.*, 2012). Additionally, MaERF9 and MaERF11 were shown to bind the *MaACS1* promoter and positively or negatively regulate its expression, and MaERF11 interacts with the *MaACO1* promoter to suppress its expression (Xiao *et al.*, 2013). In addition, MdERF2 was shown to bind the *MdACS1* promoter as a transcriptional repressor, and silencing of *MdERF2* enhanced both ethylene production and *MdACS1* expression (T. Li *et al.*, 2016).

Other plant hormones also influence ethylene biosynthesis. For example, jasmonate promotes ethylene biosynthesis by increasing the expression of *MdMYC2* to activate ethylene biosynthetic genes *MdACS1* and *MdACO1* in apple (Li *et al.*, 2017). ABA treatment triggers the expression of *SlACS* and *SlACO* genes and induces ethylene production in tomato (Zhang *et al.*, 2009). Treatment of banana with GA_3_ or the auxin hormone IAA reduces ethylene production and delays ripening (Desai & Deshpande, 1978; Lohani *et al.*, 2004).

Auxin controls a range of plant developmental processes, such as organ differentiation and the development of vascular tissues, inflorescences and fruit (Abel & Theologis, 1996; Mattsson *et al.*, 2003; Krizek, 2011). The canonical auxin signaling pathway consists of an E3 ubiquitin ligase SCF^TIR/AFB^ complex (SKP1, Cullin and auxin signaling F‐box protein), the transcriptional repressors Aux/IAAs (auxin/indole‐acetic acid proteins) and the ARF (auxin response factor) TFs (Leyser, 2018). When auxin concentrations are low, Aux/IAAs interact with ARFs and suppress their activity, so that ARFs are sequestered and are not available to trigger the transcription of their downstream genes. Conversely, higher auxin concentrations promote the interaction between Aux/IAAs and the auxin receptor TIR1 (transport inhibitor response 1), after which Aux/IAAs undergo ubiquitin‐mediated degradation and the ARFs are released to activate the expression of downstream genes (Lavy & Estelle, 2016).

The effects of auxin on ethylene biosynthesis have been reported for many horticultural corps. For example, treatment with auxin or synthetic auxin prolongs the ripening of tomato and banana fruits, and reduces the expression of ethylene‐associated genes as well as ethylene production (Lohani *et al.*, 2004; J. Li *et al.*, 2016). By contrast, IAA treatment of pear fruit induces the transcription of *PpACS1a* (*Pyrus pyrifolia* Nakai. cv Whangkeumbae) (Shi & Zhang, 2014) and treatment of plum (*Prunus salicina*) with the synthetic auxin naphthaleneacetic acid (NAA) promotes ethylene production in the fruit (El‐Sharkawy *et al.*, 2014). Studies of peach (*Prunus persica*) fruit revealed that the IAA concentration rapidly increases immediately before harvest, followed by the initiation of *PpACS1* and *PpACO1* expression and increased ethylene production, and the authors proposed that high IAA levels might induce ethylene production during ripening (Tatsuki *et al.*, 2013). An increase in endogenous auxin concentration before fruit ripening has also been observed in tomato and plum (Buta & Spaulding, 1994; El‐Sharkawy *et al.*, 2014).

Although many studies have documented the influence of auxin on ethylene biosynthesis and fruit ripening, most have only focused on the changes in gene expression or ethylene production, and have not investigated how auxin signaling genes, especially TFs, regulate ethylene biosynthetic genes. In this current study, we treated apple fruit at different developmental stages with NAA and found that this treatment strongly induced ethylene production and accelerated the onset of ripening at time points at which fruit were not able to ripen naturally. During this process, NAA‐activated *MdARF5* expression induced the expression of ethylene biosynthetic genes, and we also show that the methylation level of the *MdACS3a* promoter is important for auxin‐induced ethylene biosynthesis and fruit ripening.

## Materials and Methods

### Plant material and treatments

Apple (*M. domestica* cv Golden Delicious) fruit were collected from the Liaoning Pomology Institute (Xiongyue, China) experimental farm in 2017 and 2018. Fruit were harvested at 145 DAFB (days after full bloom) and immediately transferred to the laboratory. Harvested fruit were divided into four groups (50 fruit per group). The first group did not receive any treatment and were used as the control; the second group was submerged in 4 mM NAA (BBI Life Sciences, Shanghai, China) for 2 h; the third group was treated with 1‐MCP (1‐methylcyclopropene, an ethylene inhibitor; Fresh Doctor, Xi'an, China) for 12 h as previously described (Yue *et al.*, 2019); the fourth group was treated with 1‐MCP for 12 h, followed by the same NAA treatment. All fruits were stored at room temperature for 20 d and sampled every 5 d. Fruit harvested at 85, 95 and 115 DAFB were divided into two groups: one group was treated with 4 mM NAA, while the other group did not receive any treatment and was used as a control. The sampling regime was the same as described above. For the application of NAA on on‐tree fruit, 4 mM NAA was sprayed to the apple fruit 95 DAFB, and fruit sprayed with distilled water were used as control. All fruit were harvested 30 d after NAA spray (at 125 DAFB), and immediately transferred to the laboratory.

### Measurement of ethylene production

At each sampling time, each apple fruit was enclosed in an air‐tight container (0.86 l) with a rubber plug and maintained at room temperature for 1 h, then 1 ml of gas was collected with a 1 ml syringe for measurement of ethylene production. Ethylene was detected using a gas chromatograph (7890A, Agilent Technology, Santa Clara, CA, USA) as in Li *et al. *(2015). Ethylene production was measured for at least five fruit per sampling point.

### RNA extraction and expression analysis

Total RNA extraction and cDNA synthesis were performed according to T. Li *et al. *(2016). Gene expression was detected by quantitative reverse transcription (qRT)‐PCR on an Applied Biosystems 7500 Real‐Time PCR system, using reaction conditions and program as described by Li *et al. *(2017). The standard RT‐PCR analysis was performed as previously described (Li *et al.*, 2016). The PCR product was detected on a 1% agarose gel and imaged using a GelDoc XR System (Bio‐Rad, Hercules, CA, USA). The expression of apple *ACTIN* (EB136338) was used to confirm the equal sample loading. Fruit flesh from nine apples was sampled at each time point, and divided into three groups (three fruit per group), and fruit flesh from each group was sliced into pieces and evenly mixed for RNA extraction. RNA extracted from each group was used as one biological replicate for RT‐ and qRT‐PCR, with a total of three biological replicates. For apple fruit calli, each line of infected calli was used to represent one biological replicate, and three lines of individually infected calli were used in the experiment. All primers used for gene expression analysis are listed in Supporting Information Table S1.

### *Agrobacterium*‐mediated infiltration

For overexpression of *MdARF5* or *MdACS3a*, the CDS (coding sequence) was ligated into the pRI101 vector (TaKaRa, Shiga, Japan) to generate overexpression constructs. A partial CDS of *MdARF5* (1295–1548 bp) or *MdACS3a* (358–607 bp) was separately introduced into pRI101 in the reverse direction to generate silencing constructs using the Seamless Cloning Kit (catalog no. D7010M; Beyotime, Shanghai, China). The resulting constructs were transformed in *Agrobacterium tumefaciens* (strain EHA105). The suspension for infiltration was prepared according to T. Li *et al. *(2016). The infiltration of on‐tree apple fruit was performed as described by An *et al. *(2018) with minor modifications. Briefly, the needle of a 1 ml syringe was used to make a 2–3 cm deep pinhole on the fruit, and the suspension was then infiltrated into the pinhole with a 1 ml syringe (without the needle) using gentle pressure to make the suspension expand into the flesh as much as possible. Six to eight pinholes were made and infiltrated on each fruit. Fruit infiltrated with empty pRI101 vector were used as negative controls. The infiltrated apple fruit were harvested 5 d after infiltration and immediately transported to the laboratory. The infiltrated fruit were divided into two groups: one group did not receive any treatment and was used as control, while the other group was treated with NAA as described above. The fruit were stored at room temperature for 20 d and sampled every 5 d for investigation of ethylene production and gene expression levels. At least three fruit were used at each sampling point, and each fruit was used as one biological replicate. For silencing of *MdACS3a*, only samples of day 20 were used for detection. All chemicals were purchased from Sangon Biotech (Shanghai, China), except where otherwise indicated.

For infection of apple fruit calli (cv Orin), the *A*. *tumefaciens* EHA105 strain containing vector constructs was resuspended in 50 ml of MS medium (catalog no. M159; Phyto Technology Laboratories, Lenexa, KS, USA) containing 0.1 mM acetosyringone to generate the infection buffer. Apple calli were soaked in the infection buffer for 20 min, before being collected using a cell strainer (catalog no. CSS010040; Jet Biofil, Guangzhou, China) and cultivated on solid MS medium for 3 d. For the NAA treatment, 50 μM NAA was sprayed on the fruit calli. Each single infection of apple calli was used as one biological replicate, and three biological replicates were analyzed. The primers used are listed in Table S1.

### Chromatin immunoprecipitation (ChIP)‐PCR analysis

The *MdARF5* and *MdERF2* CDSs were ligated into the pRI101‐3xFLAG and pRI101‐GFP (green fluorescent protein) vectors, respectively, and transformed into the *A. tumefaciens* EHA105 strain, then apple fruit calli were infected as described above. The preliminary treatment of calli for the ChIP‐PCR assay was as described in Li *et al. *(2017). The ChIP assay was performed using the SimpleChip Plus Sonication Chromatin IP Kit according to the manufacturer's instruction (catalog no. 56383; Cell Signaling Technology, Danvers, MA, USA). The chromatin fragmentation was performed using an Uibra Cell VCX 150 sonicator (Sonics & Materials Inc., Newtown, CT, USA) operating at 5 s on and 8 s off for 20 cycles. The Flag (1 mg ml^–1^, catalog no. HT201‐01; Transgen, Beijing, China) and GFP (1 mg ml^–1^, catalog no. HT801‐01; Transgen) antibody were used for immunoprecipitation, and the enrichment of immunoprecipitated chromatin was analyzed by qPCR. The fruit calli were independently infected three times to generate independent lines, and each line was used for one ChIP analysis. The enrichment of DNA in each ChIP sample was used as one biological replicate, and a total of three biological replicates were analyzed. Five *MdACS3a* promoter regions, three *MdACS1* promoter regions, three *MdACO1* promoter regions, six *MdERF2* promoter regions and three *MdARF5* promoter regions were analyzed to assess their enrichment. Primers used are listed in Table S1.

### β‐Glucuronidase (GUS) activation assay

The *MdACS3a* (1250 bp from the translation start site, TSS), *MdACS1* (1213 bp from the TSS), *MdACO1* (1000 bp from the TSS) and *MdERF2* (1380 bp from the TSS) promoters were separately inserted into a GUS reporter gene vector. The *MdARF5* and *MdERF2* CDS were separately introduced into the pRI101 vector using the Seamless Cloning Kit (Beyotime) to generate the *MdARF5* and *MdERF2* overexpression vectors. All these vectors were separately transformed into *Agrobacterium* strain EHA105, before the reporter and effector constructs were co‐infiltrated into wild tobacco (*Nicotiana benthamiana*) leaves and the infiltrated tobaccos were kept in the dark at room temperature for 3 d. The measurement of GUS activity was performed according to T. Li *et al. *(2016). NAA (10 μM) was infiltrated into tobacco leaves 3 h before measurement. The primers used are listed in Table S1.

### Detection of DNA methylation levels

To verify the *MdACS3a* promoter DNA methylation level, genomic DNA was extracted from 0, 10 DAH (days after harvest) and NAA‐treated 10 DAH apple fruit samples, which were harvested at 95 DAFB. The DNA extraction was performed as described in Wang *et al. *(2013). A total of 500 ng genomic DNA was digested in a 50 μl reaction containing 2 μl of McrBC (catalog no. M0272L; New England Biolabs, Inc., Ipswich, MA, USA), 5 μl of 10× NEB Buffer 2, 0.5 μl of 100× GTP and 0.5 μl of 100× BSA, and incubated at 37°C for 18 h. The digested DNA was used directly in PCRs. A reaction without McrBC was used as a negative control. The level of DNA methylation was determined by standard PCR analysis, with the following cycling conditions: 5 min at 95°C; 35 cycles of 30 s at 95°C, 30 s at 55°C and 30 s at 72°C; and a final extension of 5 min at 72°C. The PCR products were visualized on a 1% agarose gel and photographed using an Azure C300 system (Azure Biosystems, Dublin, CA, USA). The intensity of the PCR product shift was measured using the cSeries Capture Software. A BSP (bisulfite sequence PCR) assay was performed using the EZ DNA Methylation‐Gold Kit (catalog no. D5005; Zymo Research, Irvine, CA, USA), with 200 ng DNA used for the CT conversion step, and the rest of the protocol was according to the manufacturer’s instructions. The resulting DNA was used as template for PCR. The PCR products were ligated into the pMD18‐T vector (catalog no. 6011; TaKaRa), and 12 clones for each fragment were selected for sequencing and analysis of the methylated‐cytosine, using the online kismeth software (http://katahdin.mssm.edu/kismeth/). Fruit flesh for each treatment was divided into three groups (three fruit per group), and DNA extracted from each group was used as one biological replicate for one BSP detection. A total of three biological replicates were analyzed and a total of 36 clones were sequenced for each fragment. All primers used are listed in Table S1. Other experimental methods are listed in Methods S1–S5.

## Results

### Auxin induces ethylene biosynthesis in apple fruit

The concentration of endogenous auxin during cv Golden Delicious (GD) apple fruit (*M. domestica*) development and ripening was evaluated in 2017 and 2018, and in both years endogenous IAA contents were highest at 55 DAFB, before declining rapidly to very low levels by 5 DAH. We also observed a slight increase in IAA content at 10 DAH, before ripening (Fig. S1a).

**Fig. 1:**
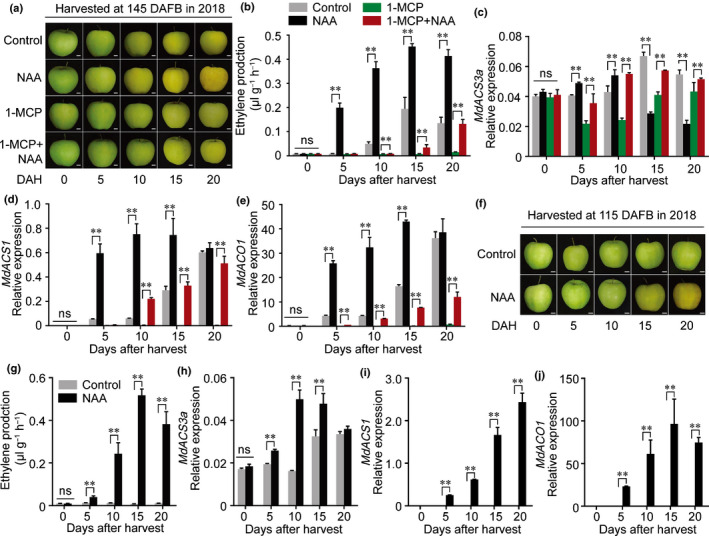
Auxin‐induced ethylene production and expression of ethylene‐related genes in apple. (a–e) Apple fruit were harvested at 145 DAFB (days after full bloom) in 2018 and treated with naphthaleneacetic acid (NAA), 1‐methylcyclopropene (1‐MCP), or with 1‐MCP followed by NAA (1‐MCP + NAA) (a), stored at room temperature for 20 d and sampled every 5 d. Fruit not receiving any treatment were used as a control. Bars, 1 cm. Ethylene production was measured (b) and the expression levels of *MdACS3a* (c), *MdACS1* (d) and *MdACO1* (e) were detected by quantitative reverse transcription (qRT)‐PCR. DAH, days after harvest. (f–j) Apple fruit were harvested at 115 DAFB and treated with NAA (f), stored at room temperature for 20 d and sampled every 5 d. Bars, 1 cm. Ethylene production was measured (g) and the expression levels of *MdACS3a* (h), *MdACS1* (i) and *MdACO1* (j) were investigated by qRT‐PCR. Fruit not receiving any treatment were used as a control. NAA, fruit treated with NAA; 1‐MCP, fruit treated with 1‐MCP; 1‐MCP + NAA, fruit treated with 1‐MCP for 12 h followed by NAA treatment. Three biological experiments from independent RNA extractions for each group of fruit were analyzed. Values represent means ± SE. Asterisks indicate significant difference as determined by a Student’s *t*‐test (**, *P* < 0.01); ns, no significant difference.

To investigate the effect of auxin on apple fruit ripening, we harvested GD apple fruit on the commercial harvest day (145 DAFB) in 2017 and 2018. Fruit were treated with NAA, 1‐MCP, or 1‐MCP followed by NAA (1‐MCP + NAA) and stored at room temperature for 20 d, then sampled every 5 d (Figs 1a,S1b). In both years, NAA treatment significantly promoted ethylene production and fruit ripening (Figs1b, S1c). The 1‐MCP treatment blocked ethylene production, but the application of NAA to 1‐MCP‐treated fruit (1‐MCP + NAA) restored ethylene biosynthesis (Figs 1b, S1c). The expression of ethylene biosynthetic genes was examined by qRT‐PCR. The expression of *MdACS6* (MDP0000133334) was not affected by the NAA treatment (Fig. S2), while the expression of *MdACS3a* (AB243060) was promoted by NAA, although it was suppressed at 15 and 20 DAH when ethylene production increased (Figs 1c, S1d). The expression of *MdACS1* (U89156) and *MdACO1* (AF030859) showed similar trends to that for ethylene production (Fig 1d,e, S1e,f).

Extending our observation that auxin induced ethylene production in apple fruit after harvest, we investigated when auxin promoted ethylene biosynthesis during fruit development. Apple fruit were harvested every 30 d from 55 DAFB, when they were not able to ripen naturally, and treated with NAA. We observed that the NAA treatment failed to induce ethylene biosynthesis at 55 or 85 DAFB (Fig. S3a,b), but strongly induced ethylene production at 115 DAFB (Figs 1f,g, S4a,b). Gene expression analysis showed that *MdACS3a* expression was enhanced by the NAA treatment (Figs 1h, S4c), while *MdACS1* and *MdACO1* expression was induced from levels that were undetectable (Figs 1i,j, S4d,e). These results indicate that auxin can induce ethylene biosynthesis even when the fruit are not able to ripen naturally.

### MdARF5 is important for auxin‐induced ethylene biosynthesis

**Fig. 2:**
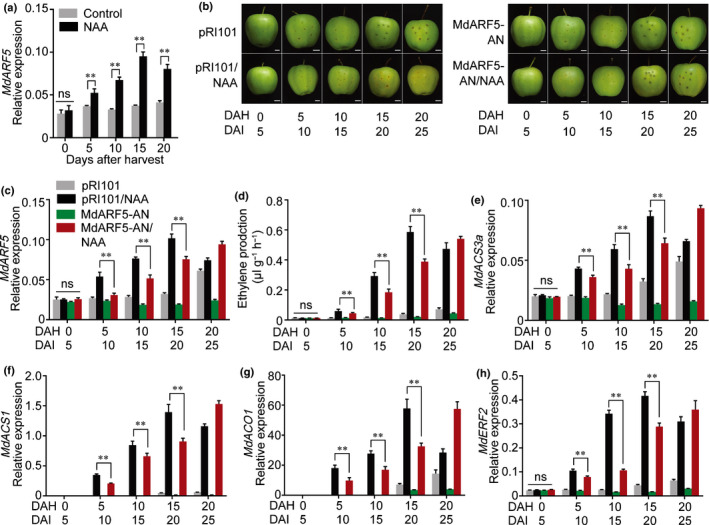
MdARF5 is required for naphthaleneacetic acid (NAA)‐induced ethylene biosynthesis in apple fruit. (a) *MdARF5* expression detected by quantitative reverse transcription (qRT)‐PCR in apple fruit treated with or without NAA. Fruit tissues were the same as in Fig. 1(f). Control, untreated fruit; NAA, NAA‐treated fruit. Three biological replicates from independent RNA extractions for each group of fruit were analyzed. (b–h) Silencing of *MdARF5* in apple fruit (MdARF5‐AN) at 110 DAFB (days after full bloom) using *Agrobacterium*‐mediated transient transformation. MdARF5‐AN fruit were harvested from the apple tree 5 d after infiltration and immediately treated with NAA (b), and stored at room temperature for 20 d. Fruit infiltrated with empty pRI101 vector were used as controls. Scale bars, 1 cm. Expression level of *MdARF5* (c) was evaluated in MdARF5‐AN fruit by qRT‐PCR to confirm successful infiltration. Ethylene production was measured (d) and the expression of *MdACS3a* (e), *MdACS1* (f), *MdACO1* (g) and *MdERF2* (h) were determined in MdARF5‐AN fruit by qRT‐PCR. Three biological experiments with independent RNA extractions were performed. Values represent means ± SE. Asterisks indicate significant differences as determined by a Student’s *t*‐test (**, *P* < 0.01); ns, no significant difference. DAH, days after harvest; DAI, days after infiltration

To explore the mechanism by which auxin induces ethylene biosynthesis in apple fruit, we compared the transcriptome, by RNA‐sequencing, of 5 DAH apple fruit samples that were harvested at 115 DAFB and treated with or without NAA. Because ARFs are the central regulator in the auxin signaling pathway (Leyser, 2018), we focused on differentially expressed ARF genes. A total of five *MdARF* genes were identified (*MdARF3*: MDP0000138860, *MdARF5*: MDP0000211459, *MdARF6*: MDP0000268306, *MdARF18*: MD01G1083400 and *MdARF19*: MDP0000876321) and we investigated their expression by qRT‐PCR to determine whether they might play a role in fruit ripening in fruit samples collected at 115 and 145 DAFB. We observed that the expression of other *MdARF*s except *MdARF5* was either unchanged or decreased during fruit ripening when harvested at 145 DAFB, indicating a negative correlation with ethylene production during fruit ripening, although their increased expression by NAA cannot explain the NAA‐induced ethylene production (Figs 1b, S5a–h). Only the *MdARF5* expression paralleled ethylene production (Figs 1b,g, 2a, S1c, S5i), and thus *MdARF5* was selected for further study. Temporal and spatial expression analysis further revealed that *MdARF5* was expressed mainly in fruit and that its expression gradually increased during fruit development and ripening (Fig. S5j,k). To analyze its subcellular localization, the *MdARF5* CDS was ligated upstream of a GFP tag under control of the *35S* promoter, and overexpressed in tobacco leaves (*N. benthamiana*) using *Agrobacterium*‐mediated transformation. Microscopy clearly showed that MdARF5‐GFP protein localized to the nucleus (Fig. S6).

To explore the putative function of *MdARF5* in ethylene production, we conducted a transient expression assay in apple fruit and apple fruit calli. The *MdARF5* CDS was cloned into the pRI101 vector under control of the *35S* promoter to generate a *35S:MdARF5* overexpression construct (MdARF5‐OE), and a partial CDS of *MdARF5* was similarly ligated into the pRI101 vector in the reverse direction to generate an *MdARF5*‐silencing vector (MdARF5‐AN). These vectors were transformed separately into *Agrobacterium* and infiltrated into on‐tree apple fruit. The infiltrated apples were harvested 5 d after infiltration (DAI), treated with NAA and stored at room temperature for 20 d (Fig. 2b). Fruit infiltrated with empty pRI101 vector were used as a control. *MdARF5* expression was significantly lower in MdARF5‐AN fruit than in control fruit (Fig. 2c), as was ethylene production (Fig. 2d). After NAA treatment, MdARF5‐AN fruit ripened more slowly, especially in the infiltrated area (Fig. 2b, at 15 and 20 DAH) and ethylene production was significantly lower than in control fruit (Fig. 2d). *MdACS3a*, *MdACS1* and *MdACO1* expression correlated with the changes in *MdARF5* expression (Fig. 2e–g). In addition, overexpression of *MdARF5* (MdARF5‐OE fruit) substantially promoted ripening, ethylene production and the expression of ethylene biosynthetic genes (Fig. S7). Moreover, we then generated three lines of *MdARF5*‐silenced apple fruit calli (MdARF5‐AN1/2/3) using *Agrobacterium*‐mediated transformation. Ethylene production and expression levels of *MdACS3a*, *MdACS1* and *MdACO1* were significantly lower in MdARF5‐AN calli compared with control calli, even after NAA treatment (Fig. S8a–e). These results indicated that MdARF5 is important for auxin‐induced ethylene biosynthesis in apple fruit.

### Auxin‐activated MdARF5 regulates the expression of ethylene biosynthetic genes

Silencing of *MdARF5* in apple fruit and apple fruit calli considerably reduced *MdACS3a*, *MdACS1* and *MdACO1* expression (Figs 2, S8a–e) and we hypothesized that MdARF5 regulates the transcription of ethylene biosynthetic genes. Promoter analysis was carried out using place (https://www.dna.affrc.go.jp/PLACE/), and several AuxREs (auxin response elements), which can be recognized by ARFs (Guilfoyle & Hagen, 2007), were identified in the *MdACS3a*, *MdACS1* and *MdACO1* promoters. We next tested whether and which part of MdARF5 could bind the *MdACS3a* promoter, targeting three parts of the protein: the N‐terminus (N) containing a B3 DNA binding domain; the middle region (M); and the C‐terminus (C), which is called the CTD (carboxy‐terminal dimerization domain) region and is in charge of forming a heterodimer with Aux/IAA proteins (Roosjen *et al.*, 2018). A yeast one‐hybrid (Y1H) assay was performed with these regions, which showed that the N‐terminus bound to the *MdACS3a* promoter (Fig. S9a). We next performed an electrophoretic mobility shift assay (EMSA) to confirm this binding. Three AuxREs were identified in the *MdACS3a* promoter, one of which was selected as the region to generate a corresponding hot probe. MdARF5 bound to the AuxRE element in the *MdACS3a* promoter, and the mobility shifted band was weakened by adding unlabeled cold competitor probe (Fig. S9b), indicating that MdARF5 binds to the *MdACS3a* promoter *in vitro*.

**Fig. 3:**
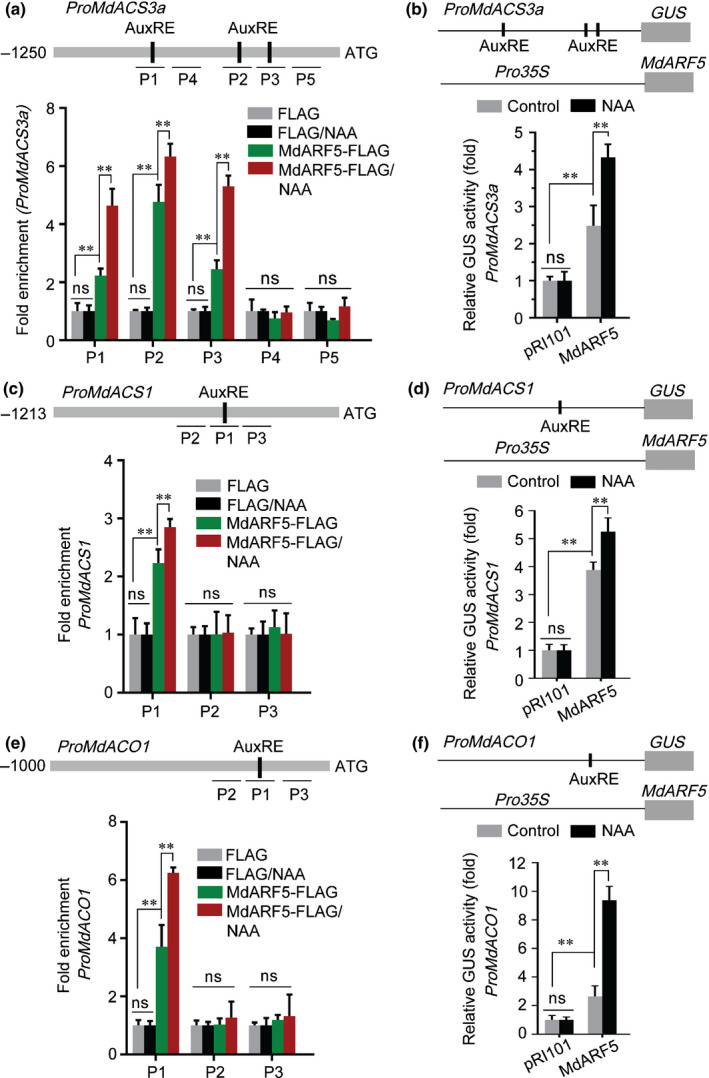
MdARF5 positively regulates ethylene biosynthetic genes through binding to their promoters. (a) ChIP (chromatin immunoprecipitation)‐PCR analysis showing MdARF5 binding to the *MdACS3a* promoter (1250 bp) *in vivo*. Cross‐linked chromatin samples were extracted from MdARF5‐FLAG‐overexpressing fruit calli treated with or without naphthaleneacetic acid (NAA) and precipitated with FLAG antibody. Eluted DNA was used to amplify sequences neighboring the AuxRE (auxin responsive element, ARF binding site) by quantitative PCR (qPCR). Five fragments (P1–P5) were analyzed. Fruit calli overexpressing the FLAG sequence alone were used as a negative control. (b) GUS (*β*‐glucosidase) activation assay showing that MdARF5 positively regulates the *MdACS3a* promoter. The MdARF5 effector vector and *MdACS3a* promoter reporter vector were co‐infiltrated into wild tobacco (*Nicotiana benthamiana*) leaves to analyze GUS activity. (c) ChIP‐PCR analysis showing MdARF5 binding to the *MdACS1* promoter (1213 bp) *in vivo*. The ChIP assay was conducted as in Fig. 3(a). Three fragments (P1–P3) were analyzed. (d) GUS activation assay showing that MdARF5 positively regulates the *MdACS1* promoter. The MdARF5 effector vector and the *MdACS1* promoter reporter vector were co‐infiltrated into wild tobacco leaves to analyze GUS activity. (e) ChIP‐PCR analysis showing MdARF5 binding to the *MdACO1* promoter (1000 bp) *in vivo*. The ChIP assay was conducted as in Fig. 3(a). Three fragments (P1–P3) were analyzed. (f) GUS activation assay showing that MdARF5 positively regulates the *MdACO1* promoter. The MdARF5 effector vector and *MdACO1* promoter reporter vector were co‐infiltrated into wild tobacco leaves to analyze GUS activity. For ChIP‐PCR, the ChIP assay was repeated three times and the enriched DNA fragments in each ChIP were used as one biological replicate for qPCR. For the GUS activation assay, three independent transfections were analyzed. Values represent means ± SE. Asterisks indicate significant differences as determined by a Student’s *t*‐test (**, *P* < 0.01); ns, no significant difference.

A ChIP‐PCR assay was used to investigate whether this binding also took place *in vivo*. The CDS of *MdARF5* was ligated upstream of 3xFLAG peptide tags under control of the *35S* promoter in the pRI101 vector, and expressed in apple fruit calli (MdARF5‐FLAG), after which ChIP‐PCR was conducted. The presence of MdARF5 enhanced the PCR‐based detection of fragments (P1, P2 and P3) containing AuxRE (Fig. 3a), indicating that MdARF5 also binds to the *MdACS3a* promoter *in vivo*. We also observed that the NAA treatment significantly enhanced this binding (Fig. 3a). We then investigated the regulation of the *MdACS3a* promoter by MdARF5 using a GUS activation assay in wild tobacco leaves. Co‐transformation with the *Pro35S:MdARF5* and *ProMdACS3a:GUS* constructs showed increased GUS activity, indicating that MdARF5 activated the *MdACS3a* promoter (Fig. 3b), and NAA treatment further enhanced *MdACS3a* promoter activity (Fig. 3b).

We next investigated the binding to, and regulation of, the *MdACS1* and *MdACO1* promoters by MdARF5. Y1H, EMSA and ChIP‐PCR analyses showed that MdARF5 bound to both promoters (Figs 3c,e, S9c–f), and GUS activation assays further demonstrated both that MdARF5 induced their promoters, and that NAA treatment enhanced the promoter activities (Fig. 3d,f).

### MdARF5 regulates *MdERF2* through binding to its promoter

**Fig. 4:**
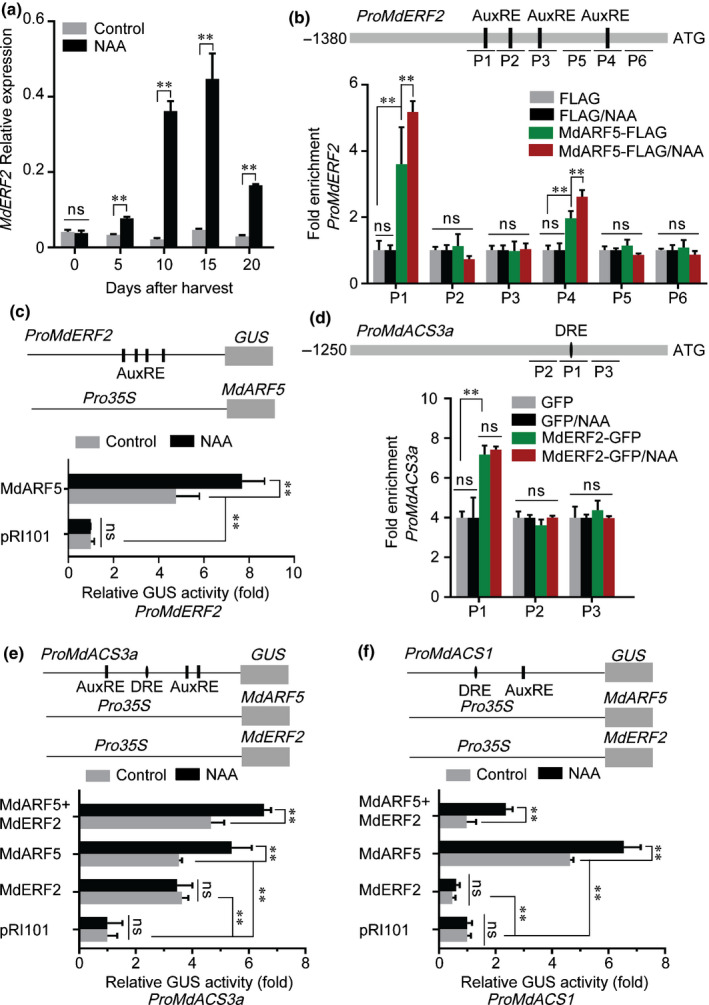
MdARF5 together with MdERF2 regulate ethylene biosynthesis. (a) The expression level of *MdERF2* was determined by quantitative reverse transcription (qRT)‐PCR in apples treated with or without naphthaleneacetic acid (NAA). Fruit tissues were the same as in Fig. 1(f). Control, untreated fruit; NAA, NAA‐treated fruit. Three biological experiments with independently extracted RNA for each group of fruit were analyzed. Values represent means ± SE. Asterisks indicate significant differences as determined by a Student’s *t*‐test (**, *P* < 0.01); ns, no significant difference. (b) ChIP (chromatin immunoprecipitation)‐PCR assay showing MdARF5 binding to the *MdERF2* promoter (1380 bp) *in vivo*. ChIP‐PCR was conducted as in Fig. 3(a). Six fragments (P1–P6) were analyzed. (c) GUS (*β*‐glucosidase) activation assay showing that MdARF5 positively regulates the *MdERF2* promoter. The MdARF5 effector vector and *MdERF2* promoter reporter vector were co‐infiltrated into wild tobacco leaves to analyze GUS activity. (d) ChIP‐PCR assay showing MdERF2 binding to the *MdACS3a* promoter (1250 bp) *in vivo*. Cross‐linked chromatin samples were extracted from MdERF2‐GFP (green fluorescent protein)‐overexpressing fruit calli, which were treated with or without NAA and precipitated with GFP antibody. Eluted DNA was used to amplify sequence neighboring the DRE‐motif (dehydration responsive element, ERF binding site) by qPCR. Three fragments (P1–P3) were analyzed. Fruit calli overexpressing the GFP sequence were used as a negative control. (e) GUS activation assay showing that MdARF5 together with MdERF2 promotes *MdACS3a* promoter activity. The MdARF5 and MdERF2 effector vectors were co‐infiltrated into wild tobacco leaves with the *MdACS3a* promoter effector vector, before analysis of GUS activity. (f) GUS activation assay showing that MdERF2 reduces *MdACS1* promoter activity promoted by MdARF5. The MdARF5 and MdERF2 effector vectors were co‐infiltrated into wild tobacco leaves with the *MdACS1* promoter effector vector to analyze GUS activity. For ChIP‐PCR, the ChIP assay was repeated three times and the enriched DNA fragments in each ChIP were used as one biological replicate for qPCR. For the GUS activation assay, three independent transfections were analyzed. Values represent means ± SE. Asterisks indicate significant differences as determined by a Student’s *t*‐test (**, *P* < 0.01); ns, no significant difference.

Previous reports have demonstrated that MdERF2 (AB288348) suppresses both the expression of *MdACS1* and ethylene production in apple (T. Li *et al.*, 2016, 2017). Based on the RNA‐seq data, we concluded that *MdERF2* was differentially expressed (Table S2), and qRT‐PCR analysis suggested that its expression was significantly promoted by NAA treatment (Fig. 4a), and was reduced in MdARF5‐silenced apple fruit and apple fruit calli (MdARF5‐AN) (Figs 2h, S8f). We next tested whether MdARF5 regulates *MdERF2* transcriptionally. A promoter analysis revealed four AuxREs in the *MdERF2* promoter, and Y1H and EMSA analyses indicated that MdARF5 bound to the AuxRE in the *MdERF2* promoter *in vitro* (Fig. S9g,h). When ChIP‐PCR was performed in MdARF5‐FLAG‐overexpressing calli, the *MdERF2* promoter P1 and P4 fragments were enriched with the presence of MdARF5 (Fig. 4b), indicating that MdARF5 also binds to the *MdERF2* promoter *in vivo*. Moreover, this enrichment was enhanced by NAA treatment (Fig. 4b).

Regulation of the *MdERF2* promoter by MdARF5 was then investigated using a GUS activation assay, which showed that MdARF5 induced the expression of *MdERF2*, and that NAA treatment strengthened the effect (Fig. 4c). Because both MdARF5 and MdERF2 are TFs, we investigated whether MdERF2 could bind the *MdARF5* promoter and regulate its expression. The CDS of *MdERF2* was ligated downstream of a GFP tag in the pRI101 vector and overexpressed in apple calli (MdERF2‐GFP), before performing ChIP‐PCR. We found that the *MdARF5* promoter region was not enriched in MdERF2‐GFP‐overexpressing calli using PCR‐based detection (Fig. S10), indicating that MdERF2 does not regulate *MdARF5* through direct binding of its promoter.

Our previous studies demonstrated that MdERF2 binds to the *MdACS1* promoter and represses its transcription (T. Li *et al.*, 2016, 2017). Here we investigated the regulation of *MdACS3a* by MdERF2. ChIP‐PCR was conducted using the MdERF2‐GFP‐overexpressing calli. We observed that the *MdACS3a* promoter was significantly enriched with the presence of MdERF2 although the NAA treatment did not enhance this enrichment (Fig. 4d), indicating that MdERF2 binds to the *MdACS3a* promoter *in vivo*. Additionally, NAA treatment did not further enhance the enrichment*.* A GUS activation assay further indicated that when a construct expressing the *MdERF2* CDS was co‐infiltrated with the *MdACS3a* promoter in wild tobacco leaves, MdERF2 induced *MdACS3a*, although it was not affected by the NAA treatment (Fig. 4e). Because both MdARF5 and MdERF2 regulated the expression of *MdACS3a* and *MdACS1*, we then investigated how MdARF5 and MdERF2 together regulated *MdACS3a* and *MdACS1* using a GUS activation assay. When MdARF5 and MdERF2 were co‐infiltrated with the *MdACS3a* promoter in tobacco leaves, *MdACS3a* promoter activity increased significantly, and NAA treatment further enhanced the activation (Fig. 4e). By contrast, when they were co‐infiltrated with the *MdACS1* promoter, promoter activity was considerably repressed compared with controls infiltrated with MdARF5 alone, and NAA treatment only partially rescued the activation (Fig. 4f). We also analyzed regulation of *MdACO1*, but we did not find any ERF binding sites on the *MdACO1* promoter (defined here as 2000 bp upstream of the TSS), and so did not study the effect of MdERF2 on the *MdACO1* promoter.

### Auxin‐induced ethylene biosynthesis is dependent on the level of *MdACS3a* promoter methylation in apple fruit

**Fig. 5:**
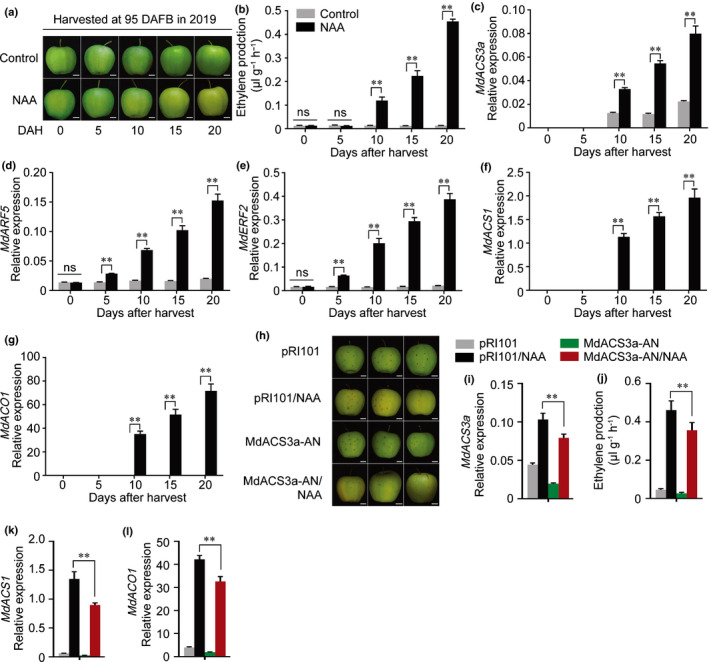
*MdACS3a* expression is crucial for auxin‐induced ethylene biosynthesis in apple fruit. (a–g) Apple fruit harvested at 95 DAFB (days after full bloom) were treated with naphthaleneacetic acid (NAA), stored at room temperature for 20 d, and sampled every 5 d (a, fruit phenotypes). Fruit not receiving any treatment were used as control. Bars, 1 cm. Ethylene production was measured (b) and the expression levels of *MdACS3a* (c), *MdARF5* (d), *MdERF2* (e), *MdACS1* (f) and *MdACO1* (g) were determined by quantitative reverse transcription (qRT)‐PCR. DAH, days after harvest. (h–l) *MdACS3a* silencing in on‐tree apple fruit (MdACS3a‐AN) at 110 DAFB performed using *Agrobacterium*‐mediated transient transformation. Fruit were harvested 5 d after infiltration and immediately treated with NAA, stored at room temperature for 20 d and sampled. Fruit infiltrated with empty pRI101 vector were used as a control (h, fruit phenotypes). Bars, 1 cm. *MdACS3a* expression (i) was determined to confirm successful infiltration as determined by qRT‐PCR. Ethylene production was measured (j) and expression levels of *MdACS1* (k) and *MdACO1* (l) were determined by qRT‐PCR. For qRT‐PCR, three biological experiments from independent RNA extractions were performed. Values represent means ± SE. Asterisks indicate significant differences as determined by a Student’s *t*‐test (**, *P* < 0.01); ns, no significant difference.

Previous studies have demonstrated that *MdACS6* and *MdACS3a* are expressed during apple fruit development, with *MdACS6* being expressed immediately after full bloom and *MdACS3a* expression initiating around 105 DAFB (Li *et al.*, 2015). We observed that NAA did not induce ethylene biosynthesis (at 55 or 85 DAFB) until 115 DAFB in apple fruit (Figs 1, S3), and that the expression of *MdACS6* was not affected by the NAA treatment (Fig. S2). We therefore hypothesized that the expression of *MdACS3a* might be important for NAA‐induced ethylene biosynthesis in early stages of apple fruit development. To test this, we harvested apple fruit at 95 DAFB, treated them with NAA and stored them at room temperature for 20 d (Fig. 5a). The NAA treatment did not induce ethylene production at 5 DAH when *MdACS3a* was not expressed (Fig. 5b,c), although *MdARF5* and *MdERF2* expression had been induced by the NAA treatment at this time point (Fig. 5d,e), and in the fruit harvested at 85 DAFB (Fig. S3c). However, once the expression of *MdACS3a* was initiated (from 10 DAH) (Fig. 5c), the NAA treatment significantly induced ethylene production (Fig. 5b). Notably, *MdACS1* and *MdACO1* expression was induced by the NAA treatment from 10 DAH when the expression of *MdACS3a* was initiated (Fig. 5f,g). In addition, we also applied NAA to on‐tree fruit at 95 DAFB. We observed that NAA‐treated fruit were ripened on‐tree at 125 DAFB and produced significantly higher ethylene than control fruit (Fig. S11).

We then silenced *MdACS3a* in apple fruit using *Agrobacterium* infiltration (Fig. 5h). *MdACS3a* expression was significantly reduced in *MdACS3a*‐silenced fruit (MdACS3a‐AN) (Fig. 5i), and ethylene production was also lower compared with control fruit (Fig. 5j). After NAA treatment, slower ripening, especially at the infiltrated areas, and lower ethylene production were observed in MdACS3a‐AN fruit than in control fruit (Fig. 5h,j). Moreover, *MdACS1* and *MdACO1* expression levels were also lower in MdACS3a‐AN fruit, even after NAA treatment, compared with control fruit (Fig. 5k,l). We overexpressed *MdACS3a* in apple fruit calli using *Agrobacterium*‐mediated transformation, which resulted in higher ethylene production and increased expression of *MdACS1* and *MdACO1* (Fig. S12). These results indicated that the expression of *MdACS3a* is important for auxin‐induced ethylene biosynthesis in apple fruit.

**Fig. 6:**
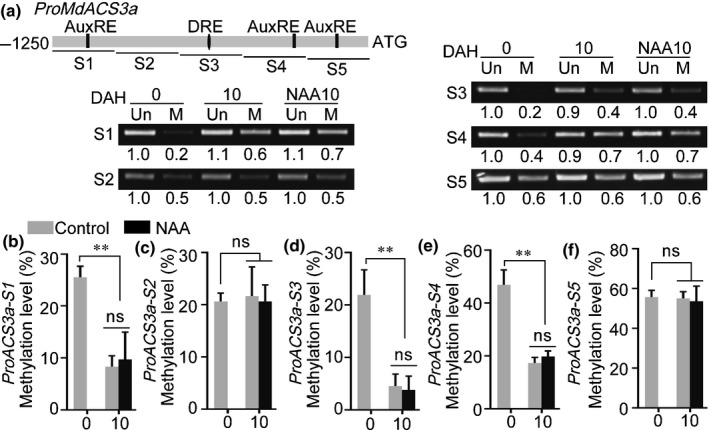
DNA methylation analysis of the *MdACS3a* promoter in apple fruit. (a) Detection of *MdACS3a* promoter methylation. Genomic DNA was extracted from the 0 and 10 DAH samples in Fig. 5(a), digested with McrBC and used as template for PCR‐based methylation detection. The *MdACS3a* promoter was divided into five fragments (S1–S5), and its methylation level was investigated by standard PCR. DNA incubated without McrBC was used as a control. Un, DNA incubated without McrBC; M, DNA incubated with McrBC; DAH, days after harvest; NAA10, fruit treated with NAA and sampled at 10 DAH. Numbers below the PCR bands indicate the shift in intensity. (b–f) *MdACS3a* promoter methylation level detected using BSP (bisulfite sequencing PCR). The same genomic DNA as in (a) was used for bisulfite modification, and the product was used as a template to amplify the five *MdACS3a* promoter regions: S1 (b), S2 (c), S3 (d), S4 (e) and S5 (f). Control, nontreated DNA; NAA, NAA‐treated DNA. DAH, days after harvest. Three biological experiments from independent DNA extractions were performed. Values represent means ± SE. Asterisks indicate significant differences as determined by a Student’s *t*‐test (**, *P* < 0.01); ns, no significant difference.

It has been reported that the level of DNA methylation can be a key factor affecting gene expression in ripening fruit (Manning *et al.*, 2006; Seymour *et al.*, 2008; Zhong *et al.*, 2013). We examined the methylation level of the *MdACS3a* promoter isolated from 0 and 10 DAH fruit samples, which were harvested at 95 DAFB and treated with NAA. The genomic DNA was digested with the McrBC enzyme, which recognizes methylated cytosine residues. After digestion, the methylation level was determined in five *MdACS3a* promoter fragments (S1–S5) using PCR. McrBC‐treated samples (M) showed brighter bands in extracts from 10 DAH fruit than from 0 DAH fruit for the S1, S3 and S4 fragments (Fig. 6a), indicating that the level of *MdACS3a* promoter methylation was lower at 10 DAH than at 0 DAH. Moreover, after NAA treatment, the brightness of the band from the NAA‐treated samples (NAA10) was similar to that in the 10 DAH samples, indicating that NAA treatment did not change the level of methylation. To confirm these results, we performed a BSP assay to measure the methylation level of the *MdACS3a* promoter. Methylation levels of the S1, S3 and S4 fragments were 25, 20 and 40%, respectively, at 0 DAH, and were reduced to 10, 5 and 20%, respectively, at 10 DAH (Fig. 6b–f). No differences were observed for fragments S2 and S5, and there were no differences between untreated and NAA‐treated samples (Fig. 6b–f). Methylation levels of the *MdACS1* and *MdACO1* promoters in the same samples as above did not show any differences (Fig. S13).

## Discussion

Treatment with exogenous auxin has been shown to increase ethylene production in pear, plum and peach fruit (Tatsuki *et al.*, 2013; El‐Sharkawy *et al.*, 2014; Shi & Zhang, 2014). Moreover, overexpression of *SlARF2* in tomato fruit leads to enhanced expression of ethylene biosynthetic genes, increased ethylene production and accelerated fruit ripening (Breitel *et al.*, 2016), while Zouine *et al. *(2014) reported that SlARF2 repressed the activity of AuxRE motif‐containing promoters, suggesting that it is a transcriptional repressor. These studies did not indicate a detailed mechanism through which auxin regulates ethylene biosynthetic genes to enhance ethylene production. Here, we determined that auxin‐activated MdARF5 induces the expression of ethylene biosynthetic genes (*MdACS3a*, *MdACS1* and *MdACO1*) by directly binding to their promoters to induce ethylene biosynthesis in apple fruit, even when the fruit is not able to ripen naturally. MdARF5 was observed to activate MdERF2 transcriptionally, and MdERF2 in turn bound to the *MdACS3a* promoter and promoted its expression, leading to elevated ethylene biosynthesis. Our findings provide detailed information regarding the effect of auxin on ethylene biosynthesis in apple fruit.

We observed that NAA significantly induced expression of *MdACS3a*, *MdACS1* and *MdACO1* (Fig. 1). Interestingly, NAA treatment did not induce ethylene biosynthesis until 95 DAFB when the expression of *MdACS3a* was initiated during storage (Fig. 5b,c), and even expression of *MdARF5* and *MdERF2*, two of its important positive regulators (Fig. 4), had already been promoted before *MdACS3a* expression (5 DAH in Figs 5d,e, S3c). We therefore suggest that there may be inhibitor(s) that act to prevent *MdACS3a* from responding to NAA. Previous reports have documented that DNA methylation of the whole genome or specific genes decrease during tomato fruit ripening (Manning *et al.*, 2006; Zhong *et al.*, 2013), and the methylation of DNA directly suppressed the binding of regulators to their downstream *cis*‐elements (Tate & Bird, 1993). Our results showed that the level of *MdACS3a* promoter methylation was higher when *MdACS3a* expression had not been initiated, wherease it was significantly reduced when *MdACS3a* was expressed (Fig. 6b–f), and NAA did not affect these methylation levels (Fig. 6b–f). These findings are consistent with a model in which the high DNA methylation level of the *MdACS3a* promoter hinders the binding of MdARF5 or MdERF2 to the *MdACS3a* promoter to activate its expression. Such a model would explain why the NAA treatment did not induce ethylene production until 95 DAFB. This also explains why the fruit produced low levels of ethylene and did not ripen at the early developmental stages when endogenous auxin levels were high (Fig. S1a). An important area of future research is determining what causes the changes in DNA methylation level of the *MdACS3a* promoter during apple fruit development*.*

Previous studies have reported that System I ethylene is produced in immature stages of climacteric fruit, whereas System II ethylene is auto‐catalyzed and produced specifically during the ripening process of climacteric fruit (Alexander & Grierson, 2002), and different *ACS* genes are responsible for System I and System II ethylene production. Barry *et al. *(2000) proposed that in tomato, ethylene production via System I (which occurs due to the activation of *SlACS1A* and *SlACS4* before System II ethylene) induces the expression of *SlACS2* and System II ethylene production. Previous studies have reported that *MdACS1* is expressed specifically during fruit ripening and it was therefore proposed to be responsible for System II ethylene production, while *MdACS3a* expression was observed before ripening (Argueso *et al.*, 2007; Schaffer *et al.*, 2007; Li *et al.*, 2015). We observed that the expression of *MdACS1* was not induced by NAA treatment until *MdACS3a* expression had initiated (Fig. 5c,f). Moreover, silencing of *MdACS3a* in apple fruit significantly reduced both *MdACS1* expression and auxin‐mediated ethylene production (Fig. 5i–l), while overexpression of *MdACS3a* had the opposite effect (Fig. S12). These results suggest an important role for *MdACS3a* in inducing ripening‐related ethylene biosynthesis (System II), and our findings support the model developed in tomato wherein the *ACS* genes expressed before System II ethylene might be responsible for producing ethylene during fruit development, which in turn promote the production of System II ethylene and fruit ripening. Our results also suggest that once *MdACS3a* expression has been initiated, the apple fruit achieve ripening competence. Thus, NAA treatment could induce the biosynthesis of ripening‐related ethylene. This suggests that *MdACS3a* could be a useful marker to indicate maturation in apple fruit.

ERFs are important regulators in ethylene signaling, and regulate the expression of *ACS* genes (Guo & Ecker, 2004). We observed that auxin promoted *MdERF2* expression through the transcriptional activator MdARF5 (Fig. 4a). Our previous studies revealed that MdERF2 acts as a transcriptional repressor and binds to the *MdACS1* promoter, suppressing its expression (T. Li *et al.*, 2016, 2017). Here, we also observed that MdERF2 suppressed the activity of the *MdACS1* promoter in a GUS activation assay (Fig. 4). This led to the question of why a transcriptional repressor was induced during auxin‐induced ethylene biosynthesis. We propose that MdERF2 might act as a buffering factor to balance the positive MdARF5 regulator and prevent apple fruit from producing too much ethylene and ripening too rapidly.

We previously reported that overexpression of MdERF2 in apple fruit can stimulate the expression of *MdACS3a* (Li *et al.*, 2015), and in this current study we found evidence that MdERF2 upregulates *MdACS3a* expression (Fig. 4e). These results indicate that MdERF2 acts as a transcriptional activator of *MdACS3a* (Fig. 4e), but also as a transcriptional repressor of *MdACS1* (Fig. 4f). This might be due to the different roles of *MdACS3a* and *MdACS1* in ethylene biosynthesis during fruit development and ripening. As mentioned above, the accumulation of ethylene, before System II ethylene is produced, might initiate System II ethylene in climacteric fruit (McMurchie *et al.*, 1972; Barry *et al.*, 2000). We previously proposed that *MdACS1* is responsible for System II ethylene biosynthesis, and that *MdACS3a* is responsible for ethylene biosynthesis before the initiation of System II ethylene production (Tan *et al.*, 2012). Moreover, MdACS3a enzyme activity is much lower than that of MdACS1 (Li *et al.*, 2015), so although NAA‐induced *MdARF5* alone could directly bind the promoter of *MdACS3a* and promote its expression (Fig. 3), it may not be enough to produce sufficient ethylene to trigger auto‐catalyzed System II ethylene production. Other TFs, such as MdERF2, may be needed to further activate the transcription of *MdACS3a* to produce more System I ethylene, thereby triggering System II ethylene production. Once the System II ethylene production is initiated, it would lead to a burst of ethylene production, resulting in rapid fruit ripening, during which MdERF2 might act as a transcriptional repressor of *MdACS1* to slow down ethylene synthesis and fruit ripening. Such a mechanism would prevent apple fruit from ripening too fast. The mechanistic basis of the opposite regulatory modes of MdERF2 regulation will be an interesting question for future research.

Several studies have documented the effect of auxin on ethylene production in horticultural crops. For example, J. Li *et al. *(2016) reported that auxin application reduced ethylene production and prolonged ripening in tomato. However, NAA treatment promoted ethylene production in plum and peach fruit (Tatsuki *et al.*, 2013; El‐Sharkawy *et al.*, 2014). It was also reported that a 100 μM IAA treatment prolonged ripening in banana (Purgatto *et al.*, 2001; Lohani *et al.*, 2004), while a 57.1 μM IAA treatment had the opposite effect (Desai & Deshpande, 1978). In the present study, in addition to using a 4 mM NAA treatment, we also investigated the effects of 0.2, 0.5, 1, 2 and 3 mM NAA applications on apple fruit harvested at 115 DAFB. We found that ethylene production was promoted under all of these conditions (data not shown), indicating that the effect of auxin on ethylene production might vary between species and function in a dose‐dependent manner. Moreover, we also treated on‐tree apple fruit using NAA, and observed that NAA accelerated on‐tree fruit ripening (Fig. S11), suggesting that NAA application could be useful in determining the period of maturation in apple.

**Fig. 7:**
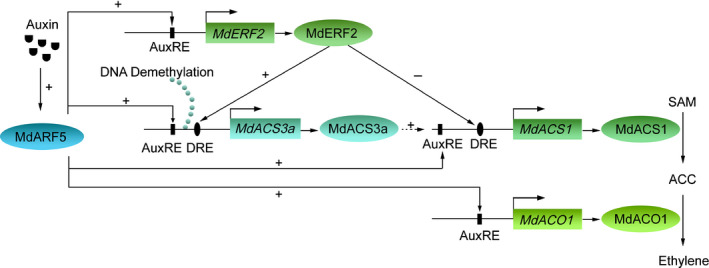
Model of auxin‐activated MdARF5 inducing ethylene biosynthesis in apple fruit. After demethylation of the *MdACS3a* promoter, its expression is initiated. Auxin‐activated MdARF5 binds to the *MdACS3a* and *MdERF2* promoters to enhance their expression, and MdERF2 then upregulates *MdACS3a* expression. Moreover, auxin‐activated MdARF5 binds to the *MdACS1* and *MdACO1* promoters and induces their expression. MdERF2 suppresses *MdACS1* expression through promoter binding in order to buffer the auxin‐activated ethylene biosynthesis. Symbols/abbreviations: ‘+’, promotion; ‘−’, suppression; solid arrow, direct regulation; dotted arrow, unclear regulation mechanism; AuxRE, auxin responsive element (ARF binding site); DRE, dehydration responsive element (ERF binding site); SAM, *S*‐adenosyl methionine; ACC, 1‐aminocyclopropane‐1‐carboxylic acid.

Taken together, our study revealed three paths for auxin‐induced ethylene biosynthesis in apple fruit: after demethylation of the *MdACS3a* promoter, *MdACS3a* expression is induced. Subsequently, auxin‐activated MdARF5 promotes the expression of *MdACS3a* directly and indirectly through MdERF2. MdARF5 also promotes the expression of *MdACS1* and *MdACO1*, and once *MdACS1* expression is initiated, MdERF2 acts as a transcriptional repressor of *MdACS1*, thereby modulating ethylene production (Fig. 7).

## Accession numbers

The raw sequence data for RNA‐seq were deposited in the NCBI Sequence Read Archive (SRA) under accession no. PRJNA544573. Sequence data from this article can be found in the Genome Database for Rosaceae (https://www.rosaceae.org/) or GenBank/EMBL libraries under accession nos. *MdARF5* (MDP0000211459), *MdARF3* (MDP0000138860), *MdARF6* (MDP0000268306), *MdARF18* (MD01G1083400), *MdARF19* (MDP0000876321), *MdACS6* (MDP0000133334), *MdACS3a* (AB243060), *MdACS1* (U89156), *MdACO1* (AF030859), *MdERF2* (AB288348) and *ACTIN* (EB136338).

## Author contributions

AW and PY designed this project and wrote the manuscript. PY performed most of the experiments. QL performed qRT‐PCR. TL and ZL provided the plant materials. HB and XL measured the ethylene content. WL and YX measured GUS activity. PY, HY and AW analyzed the data and discussed the article.

## Supporting information

Please note: Wiley Blackwell are not responsible for the content or functionality of any Supporting Information supplied by the authors. Any queries (other than missing material) should be directed to the *New Phytologist* Central Office.

**Fig. S1 **Levels of endogenous IAA, ethylene production and ethylene biosynthetic genes expression in apple fruit.
**Fig. S2** *MdACS6* expression is not affected by naphthaleneacetic acid (NAA) treatment.
**Fig. S3** Auxin treatment does not induce ethylene production and *MdACS3a* expression before 85 DAFB (days after full bloom).
**Fig. S4** Auxin induces ethylene biosynthesis in apple fruit before commercial harvest.
**Fig. S5** Expression analysis of apple *MdARF* genes.
**Fig. S6** Subcellular localization of apple MdARF5.
**Fig. S7** Overexpression of *MdARF5 *accelerates apple fruit ripening.
**Fig. S8** *MdARF5* is important for auxin‐induced ethylene biosynthesis in apple fruit calli.
**Fig. S9** MdARF5 positively regulates ethylene‐related genes of apple.
**Fig. S10** MdERF2 does not bind to the *MdARF5* promoter.
**Fig. S11** NAA treatment of on‐tree apple fruit accelerates fruit ripening.
**Fig. S12** Overexpression of *MdACS3a* increases *MdACS1* and *MdACO1* expression in apple fruit calli.
**Fig. S13** Methylation levels of* MdACS1 *and *MdACO1 *promoters in apple fruit.
**Methods S1** Measurement of endogenous IAA content.
**Methods S2** RNA‐seq analysis.
**Methods S3 **Yeast one‐hybrid (Y1H) assay.
**Methods S4** Electrophoretic mobility shift assay (EMSA).
**Methods S5** Subcellular localization analysis.

**Table S1 **Primers used in this study.
**Table S2** Differentially expressed genes between control and NAA‐treated apple fruit from RNA‐seq data.
