# Supplementary material for: Auxin‐activated MdARF5 induces the expression of ethylene biosynthetic genes to initiate apple fruit ripening
Source: New Phytol. 2020 Mar 28;226(6):1781–95. doi: 10.1111/nph.16500 (PMC7317826; doi:10.1111/nph.16500)
Supplement: Supplementary file 1 — Fig. S1 Levels of endogenous IAA, ethylene production and ethylene biosynthetic genes expression in apple fruit. Fig. S2 MdACS6 expression is not affected by naphthaleneacetic acid (NAA) treatment. Fig. S3 Auxin treatment does not induce ethylene production and MdACS3a expression before 85 DAFB (days after full bloom). Fig. S4 Auxin induces ethylene biosynthesis in apple fruit before commercial harvest. Fig. S5 Expression analysis of apple MdARF genes. Fig. S6 Subcellular localization of apple MdARF5. Fig. S7 Overexpression of MdARF5 accelerates apple fruit ripening. Fig. S8 MdARF5 is important for auxin‐induced ethylene biosynthesis in apple fruit calli. Fig. S9 MdARF5 positively regulates ethylene‐related genes of apple. Fig. S10 MdERF2 does not bind to the MdARF5 promoter. Fig. S11 NAA treatment of on‐tree apple fruit accelerates fruit ripening. Fig. S12 Overexpression of MdACS3a increases MdACS1 and MdACO1 expression in apple fruit calli. Fig. S13 Methylation levels of MdACS1 and MdACO1 promoters in apple fruit. Methods S1 Measurement of endogenous IAA content. Methods S2 RNA‐seq analysis. Methods S3 Yeast one‐hybrid (Y1H) assay. Methods S4 Electrophoretic mobility shift assay (EMSA). Methods S5 Subcellular localization analysis. [file NPH-226-1781-s001.pdf]

New Phytologist

***New Phytologist* Supporting Information**

Article title: Auxin-activated MdARF5 induces the expression of ethylene biosynthetic genes to initiate apple fruit ripening
Authors: Pengtao Yue, Qian Lu, Zhi Liu, Tianxing Lyu, Xinyue Li, Haidong Bu, Weiting Liu, Yaxiu Xu, Hui Yuan, Aide Wang*
Article acceptance date: 12 February 2020

The following Supporting Information is available for this article:

Fig. S1 Levels of endogenous IAA (indole-3-acetic acid), ethylene production and ethylene biosynthetic genes expression in apple fruit.

Fig. S2 *MdACS6* expression is not affected by naphthaleneacetic acid (NAA) treatment.

Fig. S3 Auxin treatment does not induce ethylene production and *MdACS3a* expression before 85 DAFB (days after full bloom).

Fig. S4 Auxin induces ethylene biosynthesis in apple fruit prior to commercial harvest.

Fig. S5 Expression analysis of apple *MdARF* genes.

Fig. S6 Subcellular localization of apple MdARF5.

Fig. S7 Overexpression of *MdARF5* accelerates apple fruit ripening.

Fig. S8 *MdARF5* is important for auxin-induced ethylene biosynthesis in apple fruit calli.

Fig. S9 MdARF5 positively regulates ethylene related genes of apple.

Fig. S10 MdERF2 does not bind to the *MdARF5* promoter.

Fig. S11 NAA treatment of on-tree apple fruit accelerates fruit ripening.

Fig. S12 Overexpression of *MdACS3a* increases *MdACS1* and *MdACO1* expression in apple fruit calli.

Fig. S13 Methylation levels of *MdACS1* and *MdACO1* promoters in apple fruit.

New Phytologist

Table S1 Primers used in this study (see separate file).

Table S2 Differentially expressed genes between control and NAA-treated apple fruit from RNA-seq data (see separate file).

Methods S1 Measurement of endogenous IAA content.

Methods S2 RNA-Seq Analysis.

Methods S3 Yeast one-hybrid (Y1H) assay.

Methods S4 Electrophoretic mobility shift assay (EMSA).

Methods S5 Subcellular localization analysis.

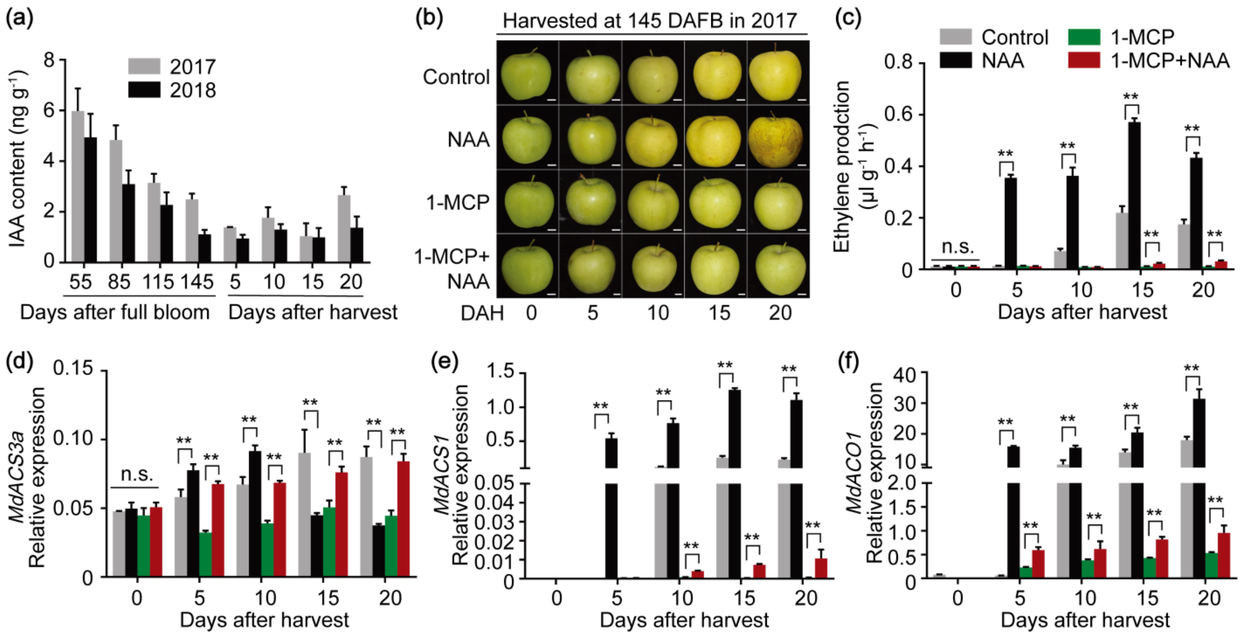

Fig. S1 Levels of endogenous IAA (indole-3-acetic acid), ethylene production and ethylene biosynthetic genes expression in apple fruit.

(a) Apple fruit were collected every 30 d from 55 DAFB (days after full bloom) during fruit development and with a final harvest at 145 DAFB in years 2017 and 2018, stored at room temperature for 20 d, and sampled every 5 d. Endogenous IAA levels were measured.
(b) to (f) Apple fruit were harvested at 145 DAFB in 2017 and treated with naphthaleneacetic acid (NAA), 1-methylcyclopropene (1-MCP), or 1-MCP followed by NAA (b, Fruit phenotypes), stored at room temperature for 20 d, and sampled every 5 d. Fruit not receiving any treatment were used as a control. Scale bars, 1 cm. The ethylene production was measured (c), and the expression levels of *MdACS3a* (d), *MdACS1* (e) and *MdACO1* (f) were detected by quantitative reverse transcription (qRT)-PCR. DAH, days after harvest. Three biological experiments from independent RNA extractions were performed. Values represent means ± SE. Asterisks indicate significant differences as determined by a Student's *t*-test (** P<0.01). n.s., no significant difference.

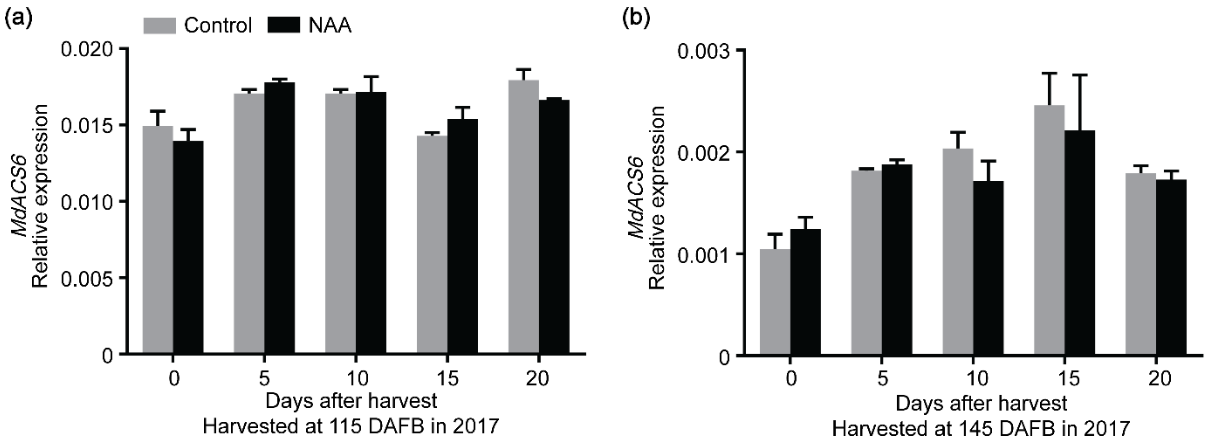

Fig. S2 *MdACS6* expression is not affected by naphthaleneacetic acid (NAA) treatment.

Apple fruit harvested at 115 DAFB (days after full bloom) (a) and Apple fruit harvested at 145 DAFB (b). All fruit were harvested in 2017 and treated with NAA, before storage at room temperature for 20 d, and sampling every 5 d. Fruit not receiving any treatment were used as a control. The *MdACS6* expression in fruit from (a) and (b) was detected by quantitative reverse transcription (qRT)-PCR. Three biological experiments from independent RNA extractions were performed. Values represent means ± SE.

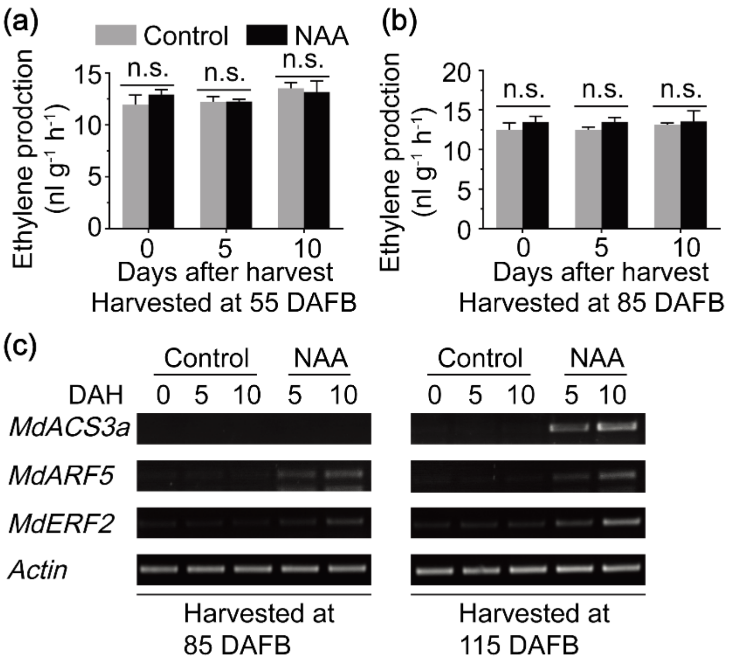

Fig. S3 Auxin treatment does not induce ethylene production and *MdACS3a* expression before 85 DAFB (days after full bloom).

Apple fruit were harvested at 55, 85 and 115 DAFB in 2017 and treated with naphthaleneacetic acid (NAA), stored at room temperature for 10 d, and sampled every 5 d. The commercial harvest was at 145 DAFB in 2017. Fruit not receiving any treatment were used as control. The ethylene production at 55 DAFB (a) and 85 DAFB (b) were measured. At least five fruit were measured at each time point. Values represent means ± SE. Significant differences was determined by a Student's *t*-test. n.s., no significant difference. The expression of *MdACS3a*, *MdARF5* and *MdERF2* (c) harvested at 85 and 115 DAFB was determined by standard PCR. *ACTIN* was used to confirm equal sample loading. DAH, days after harvest.

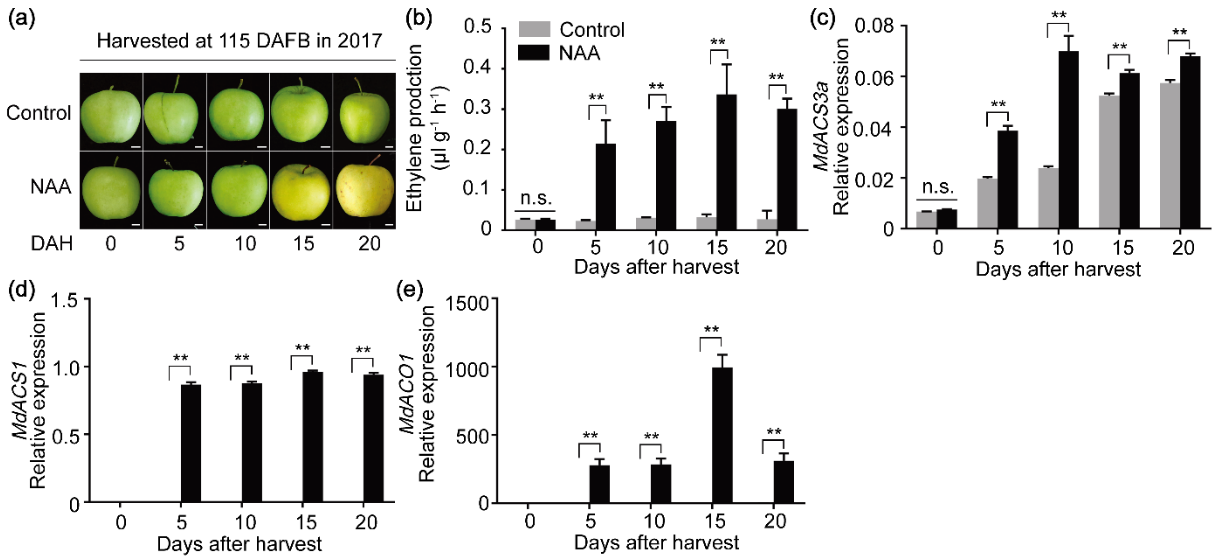

Fig. S4 Auxin induces ethylene biosynthesis in apple fruit prior to commercial harvest.

Apple fruit were harvested at 115 DAFB (days after full bloom) in 2017 and treated with naphthaleneacetic acid (NAA), stored at room temperature for 20 d, and sampled every 5 d. The commercial harvest was at 145 DAFB in 2017. Fruit not receiving any treatment were used as a control (a, Fruit phenotypes). Scale bars, 1 cm. Ethylene production was measured (b) and the expression levels of *MdACS3a* (c), *MdACS1* (d) and *MdACO1* (e) were determined by quantitative reverse transcription (qRT)-PCR. Three biological experiments from independent RNA extractions were performed. Values represent means ± SE. Asterisks indicate significant differences as determined by a Student's *t*-test (** P<0.01). n.s., no significant difference. DAH, days after harvest.

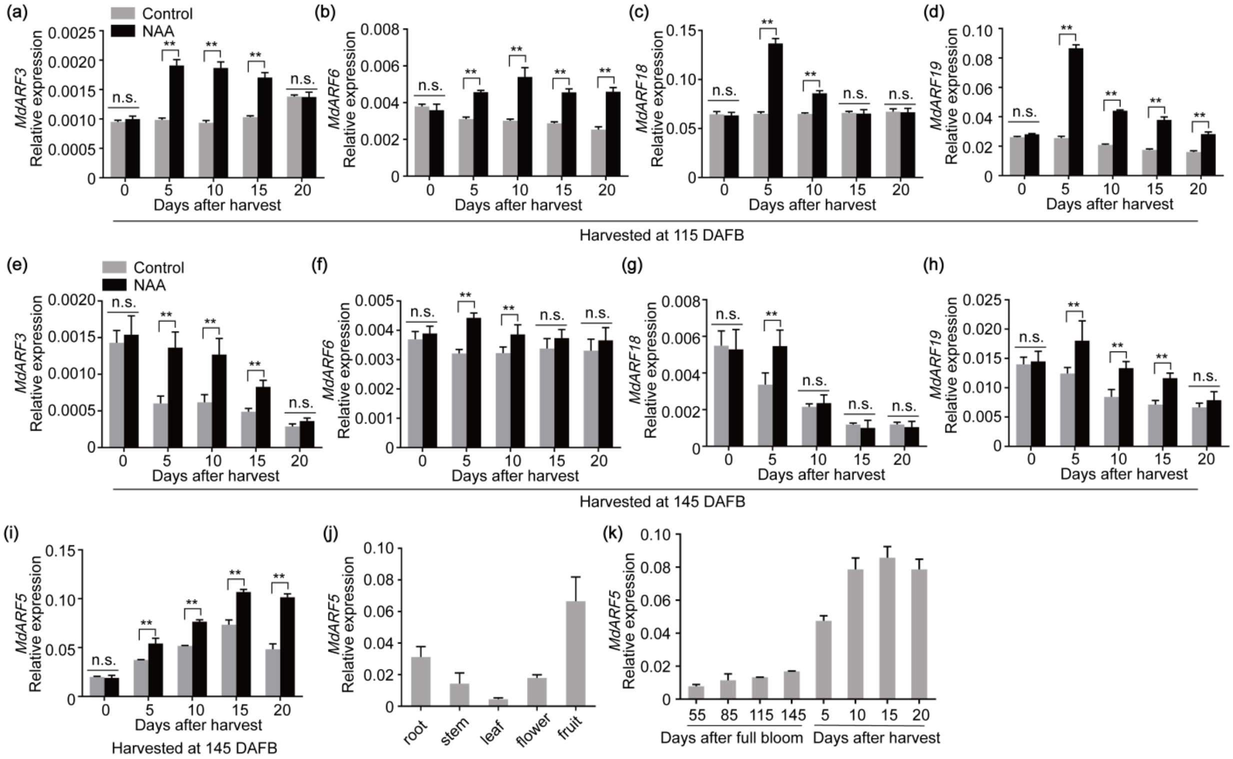

Fig. S5 Expression analysis of apple *MdARF* genes.

(a) to (d) Apple fruit were harvested at 115 DAFB (days after full bloom) in 2017 and treated with naphthaleneacetic acid (NAA), stored at room temperature for 20 d, and sampled every 5 d. Fruit not receiving any treatment were used as a control. The expression levels of *MdARF3* (a) and *MdARF6* (b), *MdARF18* (c) and *MdARF19* (d) were detected by quantitative reverse transcription (qRT)-PCR.
(e) to (i) Apple fruit were harvested at 145 DAFB in 2017 and treated with NAA, stored at room temperature for 20 d, and sampled every 5 d. Fruit not receiving any treatment were used as a control. The expression levels of *MdARF3* (e) and *MdARF6* (f), *MdARF18* (g), *MdARF19* (h) and *MdARF5* (i) were detected by qRT-PCR.
(j) *MdARF5* expression in different apple tissues by qRT-PCR. Young stems, leaves, and flowers were collected from a mature 'Golden Delicious' (GD) apple tree. Fruit were collected at 145 DAFB. Roots were collected from tissue-cultured GD.
(k) *MdARF5* expression during fruit development and ripening by qRT-PCR. Apple fruit were harvested every 30 d from 55 DAFB in 2017. The commercial harvest day was 145 DAFB. Fruit harvested at 145 DAFB were stored at room temperature for 20 d, and sampled every 5 d. Three biological experiments from independent RNA extractions were performed. Values represent means ± SE. Asterisks indicate significant differences as determined by Student's *t*-test (** $P<0.01$). n.s., no significant difference.

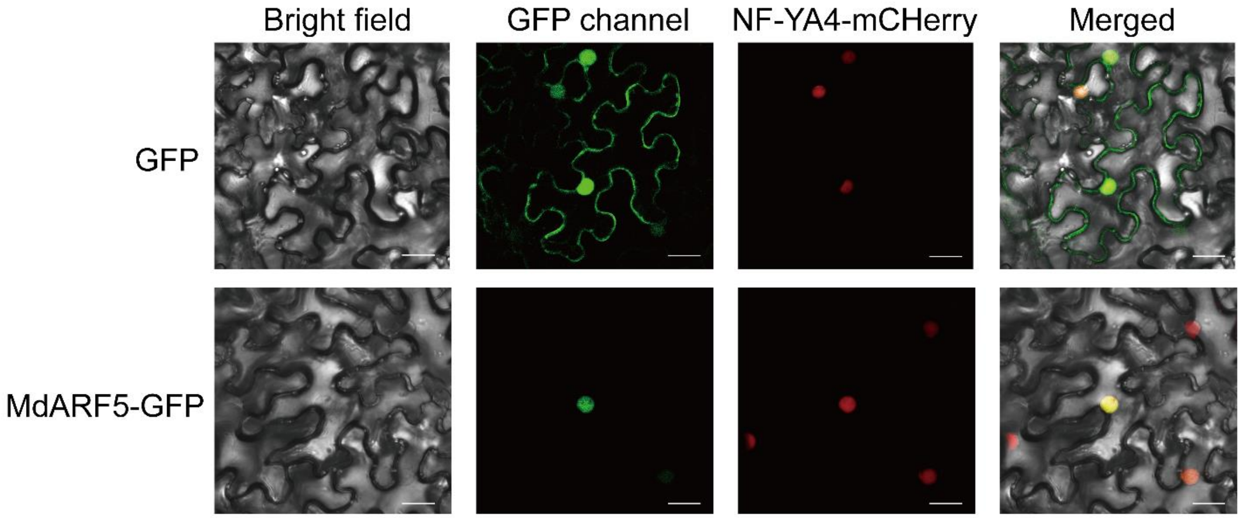

Fig. S6 Subcellular localization of apple MdARF5.

The coding sequence of *MdARF5* was ligated upstream of a GFP tag under the control of *35S* promoter in pRI101 vector, and was transiently expressed in *Nicotiana benthamiana* leaves (MdARF5-GFP). NF-YA4-mCherry was used as a nuclear marker. Transient expression of GFP alone (GFP) was used as a control. Scale bars, 25 µm.

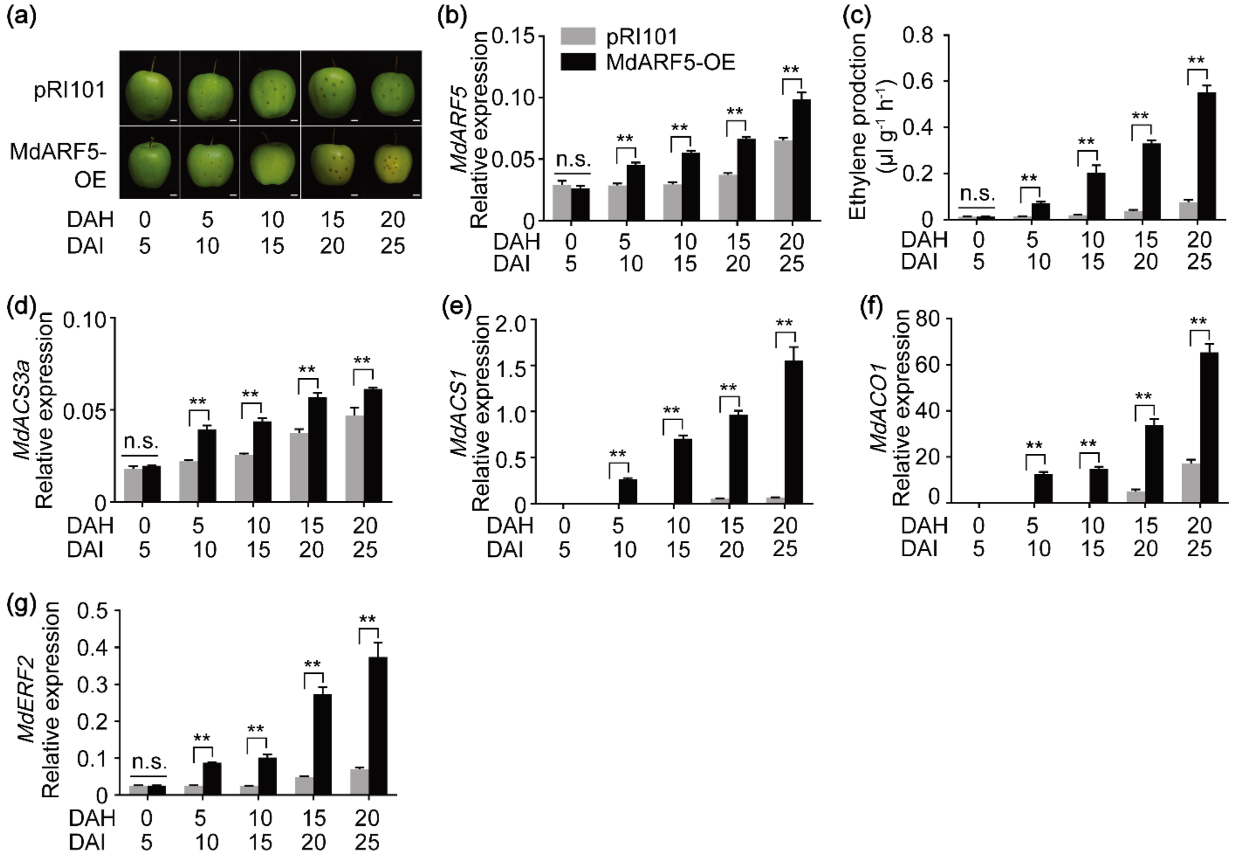

Fig. S7 Overexpression of *MdARF5* accelerates apple fruit ripening.

(a) *MdARF5* was overexpressed in apple fruit at 110 DAFB (days after full bloom) using *Agrobacterium*-mediated transient transformation (MdARF5-OE). MdARF5-OE fruit were harvested 5 d after infiltration and stored at room temperature for 20 d, and sampled every 5 d. Fruit infiltrated with empty pRI101 vector were used as a control. Scale bars, 1 cm. Expression level of *MdARF5* (b) was evaluated in MdARF5-OE fruit by quantitative reverse transcription (qRT)-PCR to confirm successful infiltration. The ethylene production was measured (c), and the expression of *MdACS3a* (d), *MdACS1* (e), *MdACO1* (f) and *MdERF2* (g) were determined in MdARF5-OE fruit by qRT-PCR. DAH, days after harvest; DAI, days after infiltration. Three biological experiments from independent RNA extractions were performed. Values represent means ± SE. Asterisks indicate significant differences as determined by a Student's *t*-test (** $P<0.01$). n.s., no significant difference.

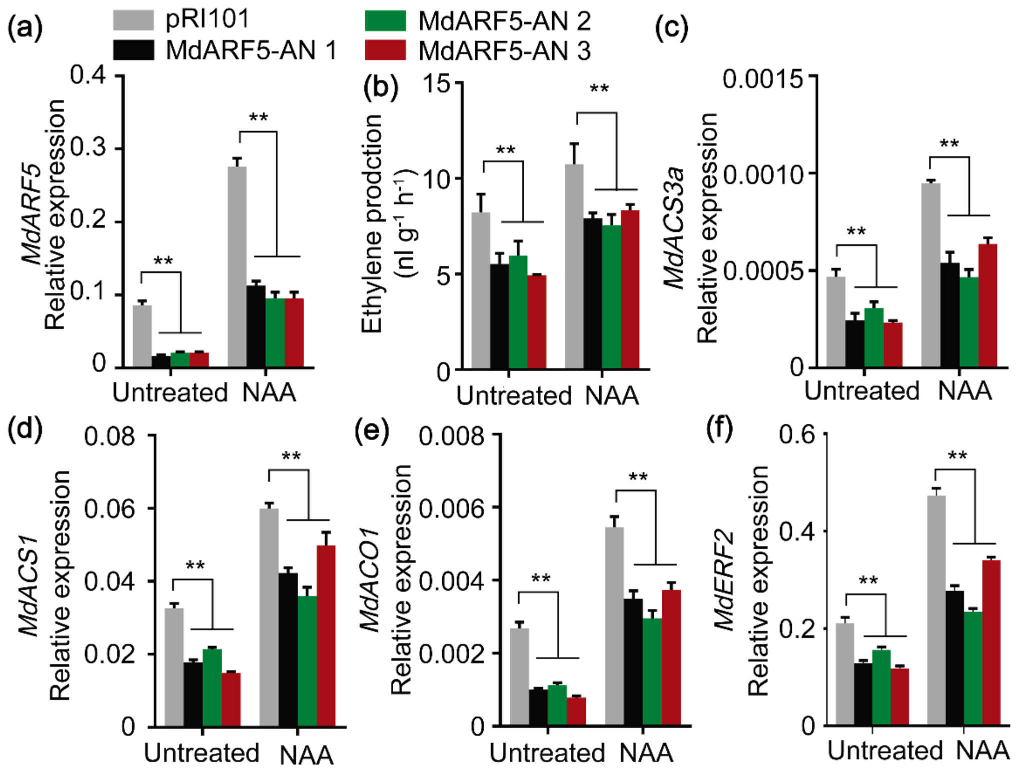

Fig. S8 *MdARF5* is important for auxin-induced ethylene biosynthesis in apple fruit calli.

MdARF5 was silenced in apple fruit calli, and three silenced lines (MdARF5-AN 1/2/3) were generated and treated with or without naphthaleneacetic acid (NAA). The expression of *MdARF5* (a) was evaluated to confirm successful silencing by quantitative reverse transcription (qRT)-PCR. The ethylene production was measured (b), and the expression of *MdACS3a* (c), *MdACS1* (d), *MdACO1* (e) and *MdERF2* (f) was determined by qRT-PCR. Calli infected with the empty pRI101 vector was used as a control. Untreated, untreated apple fruit calli; NAA, NAA treated apple fruit calli. Three biological replicates from independent RNA extractions for each treatment of calli were analyzed. Values represent means ± SE. Asterisks indicate significant difference as determined by a Student's *t*-test (** $P<0.01$).

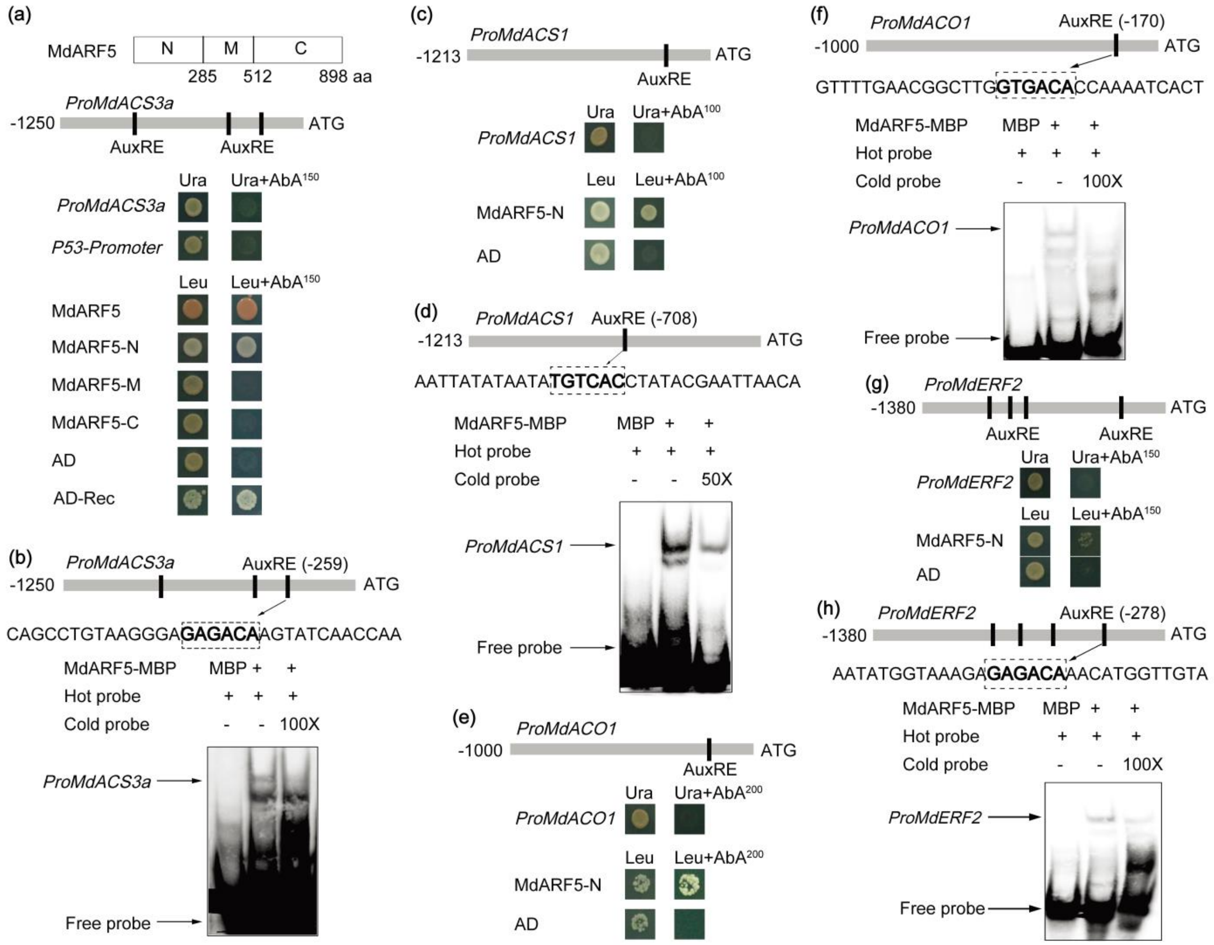

Fig. S9 MdARF5 positively regulates ethylene related genes of apple.

(a) Yeast one hybrid (Y1H) analysis showing that the MdARF5 N-terminus binds to the *MdACS3a* promoter. MdARF5 was divided into three fragments: N-terminus (1-285 aa), middle region (286-512 aa), and C-terminus (513-898 aa), which were separately co-transformed into yeast cells with the *MdACS3a* promoter. AbA (Aureobasidin A), a yeast growth inhibitor, was used as a screening marker, the basal concentration of AbA was 150 ng/ml. AD-Rec and the *P53-Promoter* were used as a positive control. The empty pGADT7 vector and the *MdACS3a* promoter were used as a negative control. AuxRE, auxin responsive element (ARF binding site).
(b) Electrophoretic mobility shift assay (EMSA) analysis showing that MdARF5 binds to the *MdACS3a* promoter. The hot probe was a biotin-labeled *MdACS3a* promoter fragment containing an AuxRE, and an unlabeled cold probe was used as the competitive probe (100 fold of that of the hot probe). MBP-tagged MdARF5 protein was purified, and the MBP protein was used as a negative control.
(c) Y1H analysis showing that the MdARF5 binds to the *MdACS1* promoter. MdARF5 was co-transformed into yeast cells with the *MdACS1* promoter. The basal concentration of AbA was 100 ng/ml. The empty pGADT7 vector and the *MdACS1* promoter were used as a negative control.

(d) EMSA showing that MdARF5 binds to the *MdACS1* promoter. The hot probe was a biotin-labeled *MdACS1* promoter fragment containing an AuxRE, and the unlabeled cold probe was used as the competitive probe (50 fold of that of the hot probe). MBP-tagged MdARF5 protein was purified, and the MBP protein was used as a negative control.
(e) Y1H analysis showing that the MdARF5 binds to the *MdACO1* promoter. MdARF5 was co-transformed into yeast cells with the *MdACO1* promoter. The basal concentration of AbA was 200 ng/ml. The empty pGADT7 vector and the *MdACO1* promoter were used as a negative control.
(f) EMSA analysis showing that MdARF5 binds to the AuxRE core of the *MdACO1* promoter. The hot probe was a biotin-labeled *MdACO1* promoter fragment containing an AuxRE, and the unlabeled cold probe was used as the competitive probe (100 fold of that of the hot probe). MBP-tagged MdARF5 protein was purified, and the MBP protein was used as a negative control.
(g) Y1H analysis showing that the MdARF5 binds to the *MdERF2* promoter. MdARF5 was co-transformed into yeast cells with the *MdERF2* promoter. The basal concentration of AbA was 150 ng/ml. The empty pGADT7 vector and the *MdERF2* promoter were used as a negative control.
(h) EMSA showing that MdARF5 binds to the AuxRE core of the *MdERF2* promoter. The hot probe was a biotin-labeled fragment of the *MdERF2* promoter containing an AuxRE, and the unlabeled cold probe was used as the competitive probe (100 fold of that of the hot probe). MBP-tagged MdARF5 protein was purified, and the MBP protein was used as a negative control.

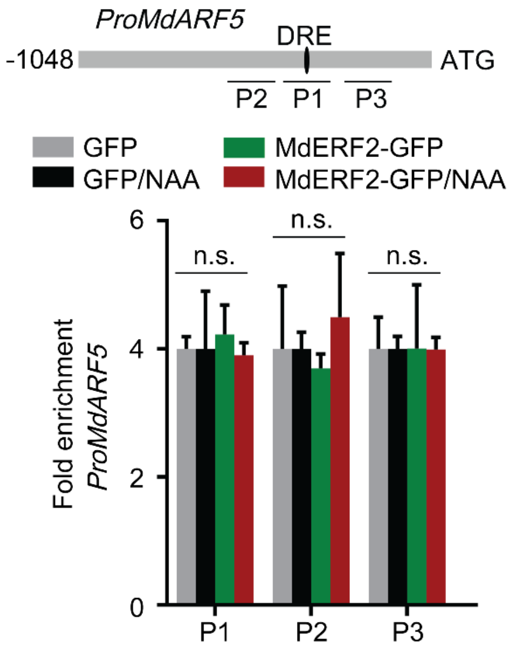

Fig. S10 MdERF2 does not bind to the *MdARF5* promoter.

A chromatin immunoprecipitation (ChIP)-PCR assay showing that MdERF2 did not bind to the *MdARF5* promoter (1,048 bp) *in vivo* in apple fruit calli. Three fragments (P1 to P3) were detected. The ChIP-PCR assay was repeated three times and the enriched DNA fragments in each ChIP were used as one biological replicate. Values represent means ± SE. Significant difference was determined by a Student's *t*-test. n.s., no significant difference.

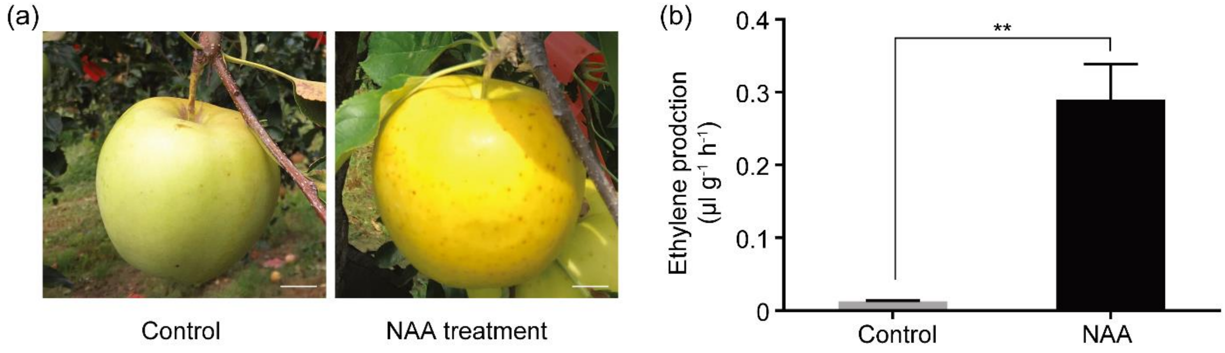

Fig. S11 NAA treatment of on-tree apple fruit accelerates fruit ripening.

NAA (naphthaleneacetic acid) was sprayed on on-tree apple fruit at 95 DAFB, and fruit were photographed (a, Fruit phenotypes) and harvested 30 d after spray. The ethylene production was measured immediately after harvest (b). On-tree fruit spayed with distilled water was used as control. Scale bars, 1 cm. At least 5 fruit were used for ethylene measurement. Values represent means ± SE. Asterisks indicate significant difference as determined by a Student's *t*-test (** $P<0.01$).

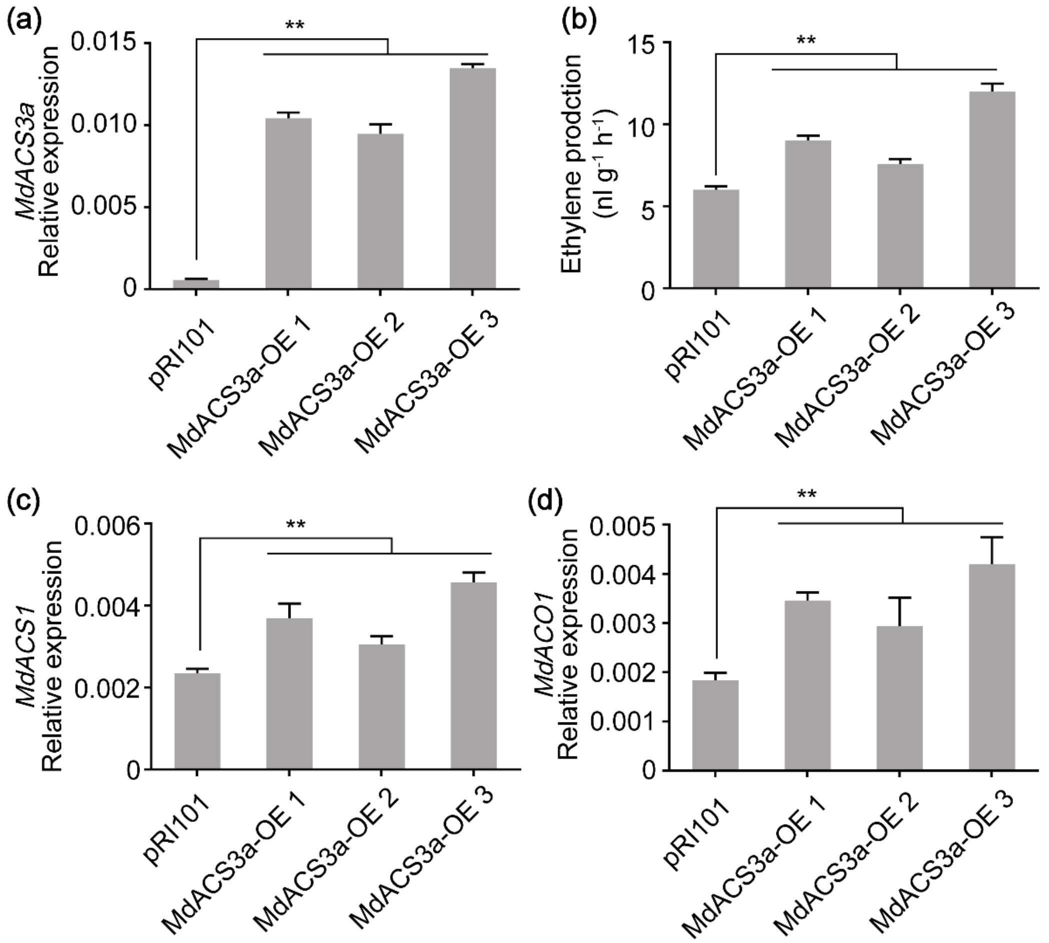

Fig. S12 Overexpression of *MdACS3a* increases *MdACS1* and *MdACO1* expression in apple fruit calli.

MdACS3a was overexpressed in apple fruit calli and three overexpression lines were generated (MdACS3a-OE 1/2/3). The expression of *MdACS3a* (a) was evaluated to confirm successful infiltration by quantitative reverse transcription (qRT)-PCR. The ethylene production was measured (b) and the expression levels of *MdACS1* (c) and *MdACO1* (d) was determined by qRT-PCR. Calli infected with empty pRI101 vector were used as a control. Three biological experiments from independent RNA extractions were performed. Values represent means ± SE. Asterisks indicate significant differences as determined by a Student's *t*-test (** $P<0.01$).

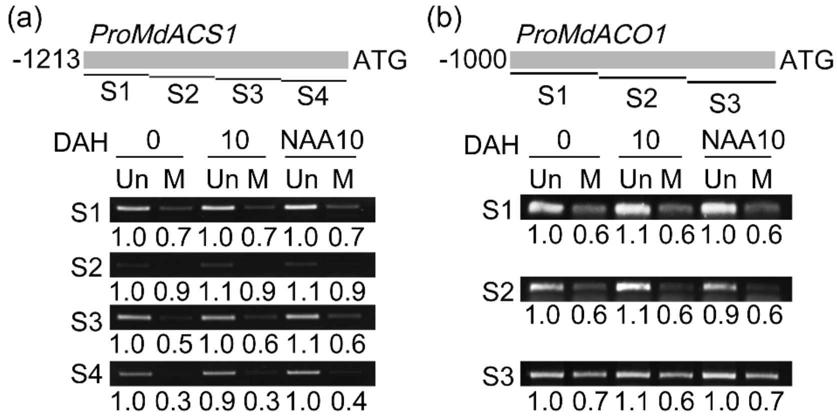

Fig. S13 Methylation levels of *MdACS1* and *MdACO1* promoters in apple fruit.

Apple fruit harvested at 95 DAFB (days after full bloom) were treated with naphthaleneacetic acid (NAA), stored at room temperature for 20 d, and sampled every 5 d. The *MdACS1* (a) or *MdACO1* (b) promoter was divided into four (S1-S4) or three (S1-S3) fragments, and the methylation was investigated by PCR. DNA incubated without McrBC was used as a control. Un, DNA incubated without McrBC; M, DNA incubated with McrBC. DAH, days after harvest; NAA10, fruit treated with NAA and sampled at 10 DAH. The numbers under the PCR bands indicate the shift intensity.

Methods S1 Measurement of endogenous IAA content.

At each sampling time, apple fruit samples were divided into three groups (3 fruit per group), and fruit flesh from each group was evenly mixed for to measure IAA content. The IAA content was measured on an UPLC/MS system (ACQUITY UPLC XEVO TQD, Waters, USA) according to Yue *et al*. (2019). The IAA content of fruit from each sample group was used as one biological replicate and at least three biological replicates were analyzed.

Methods S2 RNA-seq analysis.

Fruit samples at 5 DAH, with or without naphthaleneacetic acid (NAA) treatment at 115 DAFB, were subjected to RNA-Seq analysis. A total of 1 μg of RNA was used for generating sequencing libraries using the NEBNext Ultra™ RNA Library Prep Kit for Illumina (NEB). mRNA was generated from total RNA using poly-T oligo-attached magnetic beads (NEB). Fragmentation was carried out using divalent cations under elevated temperature in 5x NEBNext First Strand Synthesis Reaction Buffer (NEB). First strand cDNA was synthesized using random hexamer primers and M-MuLV Reverse Transcriptase (NEB), while subsequent second strand cDNA synthesis was performed using DNA Polymerase I and RNase H (NEB). To select cDNA fragments with an approximate length of 240 bp, the library fragments were purified using an AMPure XP system (Beckman Coulter, Beverly, USA). Subsequently, 3 μl of USER Enzyme (NEB) was incubated with the cDNA at 37°C for 15 min followed by 5 min at 95°C before PCR, and then PCR was performed using the Phusion High-Fidelity DNA polymerase (NEB), Universal PCR primers and Index (X) Primer. Finally, PCR products were purified (AMPure XP system) and library quality was assessed using an Agilent Bioanalyzer 2100 system. RNA sequencing was performed by the Biomarker Biotech Company (www.biomarker.com.cn), and RNA-Seq data analysis was performed as previously described (Huang *et al.*, 2014). Apple fruit samples from each treatment were divided into three groups (three fruit per group), and fruit flesh from each group were sliced into pieces and evenly mixed for RNA extraction. RNA extracted from each group represented one biological replicate, and a total of three biological replicates were analyzed. The FPKM (fragments per kb per million reads) method was used to calculate the rate of differentially expressed genes (DEGs). DEGs with a log2 (FC) value ≥ 1.5 were identified from the RNA-Seq data. The raw sequence data were deposited into the NCBI Sequence Read Archive (SRA) under the accession number PRJNA544573.

Methods S3 Yeast one-hybrid (Y1H) assay.

The coding sequence (CDS) of *MdARF5* was analyzed using the NCBI Conserved Domains analyzing tool (https://www.ncbi.nlm.nih.gov/Structure/cdd/wrpsb.cgi), and was divided into three fragments: N-terminal (1-285 amino acid, aa), middle region (286-512 aa) and C-terminal region (513-898 aa). The *MdARF5* CDS and the above three fragments were cloned and inserted into the pGADT7 vector (Clontech), respectively. The promoters of *MdACS3a* (1,250 bp from the translation start site, TSS), *MdACS1* (1,213 bp from the TSS), *MdACO1* (1,000 bp from the TSS) and *MdERF2* (1,380 bp from the TSS) were ligated into the pAbAi vector (Clontech), respectively. The Y1H assay was performed using the Matchmaker Gold Yeast One-Hybrid Library Screening

System (Clontech) according to the manufacturer's instruction. All primers used are listed in Supporting Information Table S2.

Methods S4 Electrophoretic mobility shift assay (EMSA).

The *MdARF5* CDS was inserted into the pMAL-C2X vector (catalog no. N8108S, NEB, USA) using the *Eco*RI and *Sal*I restriction sites to generate a construct encoding MdARF5-MBP fusion protien, which was transformed into *Escherichia coli* BL21 (DE3) cells (catalog no. CD601-01, Transgen, China). The cells were then cultivated in 200 ml LB medium (1% tryptone, 0.5% yeast extract and 1% (w/v) NaCl, 50 μg ml^{-1} ampicillin) for 180 rpm at 37°C until the cell culture reached OD=0.4. The cells were then centrifuged at 10,000 g for 5 min, and the resulting pellet was re-suspended in 200 ml LB medium and 0.1 mM IPTG (isopropyl-β-D-thiogalactopyranoside, catalog no. 9030, TAKARA, Japan), and incubated at 23°C for 12 h, shaking at 180 rpm. The purification of the MdARF5-MBP protein was performed as previously described (Li *et al.*, 2016). The oligonucleotide probes were synthesized and biotin-labeled (Sangon Biotech, China). The 3' biotin end-labeled double-stranded DNA was prepared by heating at 95°C for 5 min, sustaining the temperature at 72°C for 20 min and then slowly cooling to room temperature. The sequences of the biotin-labeled probes are shown in Supporting Information Fig. S9. EMSA analysis was performed as previously described (Li *et al*., 2017). All chemicals, except as otherwise indicated, were purchased from Sangon Biotech (China). All primers used are listed in Supporting Information Table S1.

Methods S5 Subcellular localization analysis.

The *MdARF5* coding sequence was ligated upstream of a GFP tag under the control of *35S* promoter in the pRI101 vector (TaKaRa) to form *Pro35S:MdARF5:GFP* construct, *Pro35S:GFP* alone was used as a control. NF-YA4-mCherry was used as nuclear marker (Zhang *et al*., 2019). The constructs were transformed into the *A. tumefaciens* EHA105 strain, and infiltrated into wild tobacco (*N. benthamiana*) leaves, and kept at room temperature for three days. The fluorescence was observed using a fluorescence microscope under a confocal microscope (TCS SP8, Leica, Germany). All transient expression assays were performed at least three times. All primers used are listed in Supporting Information Table S1.

References:

Huang G, Li T, Li X, Tan D, Jiang Z, Wei Y, Li J, Wang A. 2014. Comparative transcriptome analysis of climacteric fruit of Chinese pear (Pyrus ussuriensis) reveals new insights into fruit ripening. *PLoS One* **9**(9): e107562.

Li T, Jiang Z, Zhang L, Tan D, Wei Y, Yuan H, Li T, Wang A. 2016. Apple (Malus domestica) MdERF2 negatively affects ethylene biosynthesis during fruit ripening by suppressing MdACS1 transcription. *Plant Journal* **88**(5): 735-748.

Li T, Xu Y, Zhang L, Ji Y, Tan D, Yuan H, Wang A. 2017. The Jasmonate-Activated Transcription Factor MdMYC2 Regulates ETHYLENE RESPONSE FACTOR and Ethylene Biosynthetic Genes to Promote Ethylene Biosynthesis during Apple Fruit Ripening. *Plant Cell* **29**(6): 1316-1334.

Yue P, Wang Y, Bu H, Li X, Yuan H, Wang A. 2019. Ethylene promotes IAA reduction through PuERFs-activated PuGH3.1 during fruit ripening in pear (Pyrus ussuriensis). *Postharvest Biology and Technology* **157**.

Zhang S, Feng M, Chen W, Zhou X, Lu J, Wang Y, Li Y, Jiang CZ, Gan SS, Ma N, et al. 2019. In rose, transcription factor PTM balances growth and drought survival via PIP2;1 aquaporin. *Nat Plants* **5**(3): 290-299.
